# Global Transcriptome Analysis Reveals the Molecular Mechanism Underlying Seed Physical Dormancy Formation in *Medicago sativa*

**DOI:** 10.3390/genes16121438

**Published:** 2025-12-01

**Authors:** He Li, Xiaoying Kang, Xu Li, Feng Yuan, Zeng-Yu Wang, Maofeng Chai

**Affiliations:** 1 Shandong Key Laboratory for Germplasm Innovation of Saline-Alkaline Tolerant Grasses and Trees, College of Grassland Science, Qingdao Agricultural University, Qingdao 266109, China; 2 Hinngan League Academy of Agricultural and Animal Husbandry Sciences, Ulan Hot 137400, China; 3 Inner Mongolia Pratacultural Technology Innovation Center Co., Ltd., Hohhot 010070, China

**Keywords:** alfalfa, seed, physical dormancy, transcriptome analysis

## Abstract

Seed physical dormancy, also known as hard-seededness, is a characteristic commonly found in higher plants, which functions to prevent water and oxygen from passing through the impermeable seed coat. **Background**: Notably, seed dormancy has emerged as a critical factor in the domestication of leguminous plants. Alfalfa (*Medicago sativa* L.) is a globally cultivated high-quality legume forage crop, while the seeds from different varieties maintain varying degrees of hard-seededness. However, the molecular mechanisms underlying physical dormancy in alfalfa seeds remain poorly understood. In particular, the regulatory mechanisms at the transcriptomic level remain unclear, which has hindered the breeding process of varieties with low hard-seededness. **Methods**: In this study, we performed global transcriptome analysis to discover the genes specifically expressed in the alfalfa seed coat and provide insights into alfalfa seeds’ physical dormancy domestication traits. RNA sequencing was performed on various alfalfa tissues, including roots, stems, leaves, flowers, and seed coats. **Results**: This analysis led to the identification of 4740 seed coat-specific expressed genes, including key genes such as *KNOX4* (a class II KNOTTED-like homeobox gene), *qHs1* (encoding endo-1,4-β-glucanase), *GmHs1-1* (encoding a calcineurin-like metallophosphoesterase), and *KCS12* (β-ketoacyl-CoA synthase). In addition, several seed coat-specific transcription factor families were identified, including ERF, B3, and NAC, among others. Furthermore, a comparison of gene expression profiles between seeds with and without physical dormancy revealed 60 upregulated and 197 downregulated genes associated with physical dormancy. Crucially, functional enrichment analysis demonstrated that these genes are predominantly associated with lipid metabolism pathways, particularly those involved in the formation of “monolayer-surrounding lipid storage bodies.” **Conclusions**: This key finding suggests that the establishment of physical dormancy is closely linked to the biosynthesis and deposition of specialized lipid-based layers in the seed coat, which likely constitute the primary barrier to water penetration. Our study thus provides fundamental insights and a valuable genetic resource for future functional studies aimed at deciphering and manipulating physical dormancy in alfalfa.

## 1. Introduction

Alfalfa (*Medicago sativa* L.) is the most globally cultivated forage legume because of its high protein content, nutritional quality, adaptability to different environments, rich biomass production, and drought tolerance [1,2]. The global cultivation area is approximately 30 million ha and is mainly concentrated in the United States (30%), Europe (25%), and Argentina (23%) [3,4]. In the United States, alfalfa is a major forage legume with an average annual value of more than USD 8 billion [5]. Another advantage of alfalfa is its potential for the production of ethanol and other bioproducts [6]. Despite its promise, alfalfa has not been sufficiently adapted partly due to lack of selection during seed physical dormancy domestication.

Plant domestication empowers the use of wild plants to meet essential human needs, including food, fiber, medicine, and energy [7,8]. Seed dormancy directly affects seed germination and dispersal [9]; thus, this trait is critical in legume domestication [10]. Seed dormancy refers to an adaptive trait in which a seed can increase the period during which it can maintain viability but does not germinate. Seed dormancy under different environmental conditions is categorized into three types [11]: (1) morphological dormancy: the embryo has not yet fully developed; (2) physiological dormancy: the inner composition of the seed components prevents embryonic growth and seed germination until the seed undergoes chemical changes (e.g., abscisic acid and gibberellin metabolism); and (3) physical dormancy: the impermeable cuticle and dense palisade cell layer in the seed coat prevent viable embryos from interacting with external water and oxygen [12].

Physical dormancy, also known as hard-seededness, is an adaptive trait widespread in vascular plants and commonly observed in legumes [13,14]. After testing more than 260 species of leguminous plants, up to 85% of them were found to have seeds carrying hard-seededness traits. Physical dormancy has also been found in other families, such as Malvaceae and Chenopodiaceae [15]. In seeds with hard-seededness, the palisade cell layer becomes impermeable to water and oxygen and covers the complete cuticle, keeping the embryo alive for long periods under unfavorable conditions [16]. Only by cutting through the dense cuticle covering the outermost layer of the embryo can water and oxygen enter the seed, causing it to swell and sprout [17,18]. This seed coat impermeability is a key mechanism driving physical dormancy, and it is more prevalent in wild legumes: in natural environments, many wild legume seeds fail to germinate properly unless exposed to specific humidity and temperature conditions or subjected to seed coat abrasion [19].

In agricultural production, seeds with hard-seededness can result in less or delayed germination and increased seed use, ultimately increasing the agricultural costs. Therefore, breaking dormancy is essential for efficient and sustainable agricultural production. In the artificial domestication of soybeans, seeds with reduced hard-seededness have been selected. The reason for this selection is that when cooking legume varieties, seeds without hard-seededness are more likely to absorb water than seeds with hard-seededness, thereby improving the cooking quality [20,21]. A single gene, *GmHs1-1* (encoding a calcineurin-like metallophosphoesterase), was found to control the impermeability of wild soybean seed coats [22]. This gene encodes a calcineurin-like metallophosphoesterase transmembrane protein that is mainly expressed in the Malpighian layer (historically named for Marcello Malpighi, corresponding to the palisade/macrosclereid cell layer in the seed coat of leguminous plants) of the seed coat [23,24]. Another gene *GmqHs1* (encoding endo-1,4-β-glucanase) encodes an endonuclease β-1,4-glucanase, and a single nucleotide polymorphism in this gene leads to a mutation in the substrate-binding portion of the enzyme, resulting in reduced or eliminated affinity for substrates. Consequently, this mutation breaks down the hard-seededness of soybean seeds. In the model legume *Medicago truncatula* L., forward genetics was utilized to screen a large population of transposable element of *Nicotiana tabacum* cell type 1 (*Tnt1*) mutants for the gene *KNOX4* (a class II KNOTTED-like homeobox gene) that controls hard-seededness [25]. Recent studies have further expanded the understanding of the molecular regulatory network of physical dormancy in *M. truncatula*, demonstrating that multiple genes synergistically regulate the synthesis of seed coat cuticular lipids and impermeability. For instance, ANTHOCYANIDIN REDUCTASE (*ANR*) was identified as a novel regulator of physical dormancy, which modulates the accumulation of flavonoids and very-long-chain fatty acids (*VLCFAs*) in the seed coat to maintain seed impermeability [26]. Further investigation revealed that β-ketoacyl-CoA synthase (*KCS12*), as the downstream target gene of the *KNOX4*, was significantly downregulated in a *knox4* mutant. As a result, its mutant seeds increased water absorption [27]. This analysis revealed that *M. truncatula* produces ultra-long-chain lipids in the seed coat to control physical dormancy through the molecular mechanism by which *KNOX4-KCS12* controls physical dormancy in seeds.

Hard-seededness in alfalfa displays genetic variation and is also influenced by the local growth environment. Factors affecting hard-seededness include plant genetic variation and other factors such as the relative humidity, seed maturity, and post-harvest processing [28]. After screening over 3000 alfalfa genotypes from diverse environments, hard-seededness was found to be a highly heritable trait [29]. Differences in alfalfa varieties and growing environments, as well as the length of storage, can cause differences in hard-seededness. Additionally, alfalfa is an allotetraploid plant and self-incompatible; thus, the genetic background of each seed may differ, making studying physical dormancy difficult [30].

Although breakthroughs have been achieved in understanding the molecular mechanisms of seed physical dormancy in legume species such as soybean (*Glycine max*) and *M. truncatula*—for example, *GmHs1-1* regulates seed coat impermeability in soybean, and the *KNOX4-KCS12* pathway controls very-long-chain lipid synthesis in *M. truncatula* [22,27]—*M. sativa*, as an allopolyploid and self-incompatible species, has far greater genomic complexity than diploid model legumes. Additionally, significant variations in hard-seededness rates (20–80%) exist among different *M. sativa* cultivars [31], resulting in unclear key regulatory genes, pathways, and transcriptional regulatory networks underlying seed physical dormancy in this species. Two core research gaps currently persist: first, there is a lack of systematic screening for seed coat-specific expressed genes in *M. sativa*, making it impossible to distinguish exclusive regulatory modules for seed coat development versus dormancy formation; second, transcriptomic differences between hard and non-hard seeds have not been linked to key physiological processes such as lipid metabolism and cell wall synthesis, hindering the identification of functional genes applicable to breeding. Wen et al. proposed that anthocyanin reductase plays a role in seed hardening [26]. Wang et al. employed transcriptomic analysis to identify differential expression of genes associated with fatty acid synthesis and degradation during late seed development [32]. In 2023, Wang et al. further demonstrated through transcriptomic analysis that seed dormancy is also linked to abscisic acid (ABA) [33].

Elucidating the transcriptomic mechanism of seed dormancy in *M. sativa* holds critical practical significance for breeding and domestication. On one hand, hard seeds of *M. sativa* lead to low field germination rates, uneven seedling establishment, and a 15–20% increase in seed usage costs [29]. Mining dormancy-related genes via transcriptomics enables the development of molecular markers for the assisted selection of low-hard-seededness cultivars, thereby shortening the breeding cycle. On the other hand, as an important species in legume forage domestication, the genetic regulatory rules of dormancy traits in *M. sativa* can provide references for the domestication of other legume crops (e.g., V.villosa, *Trifolium* spp.), promoting efficient production across the entire legume forage industry. Recent studies on closely related legume species further confirm the value of this research direction: in mungbean (*Vigna radiata*), the class II *KNOX* gene *KNAT7-1* affects seed hard-seededness by regulating cuticular lipid synthesis, and transcriptomic data show that this gene is co-expressed with 12 lipid transfer protein (*LTP*) genes [34]; in hairy vetch (V.villosa), the expression levels of genes related to “monolayer-surrounding lipid storage bodies” (e.g., *LACS*, *KCS*) in the seed coats of hard seeds are 2.3–3.5 times higher than those in non-hard seeds, and this expression is regulated by the *ERF* transcription factor family. These findings suggest that seed coat-specific expression of lipid metabolism-related genes may be a conserved mechanism for dormancy formation in legumes, yet the expression patterns and regulatory relationships of such genes in *M. sativa* have not been reported.

In this study, we aimed to investigate the regulatory mechanisms underlying physical dormancy in alfalfa seeds and screen for key genes. We screened genes specifically expressed in the alfalfa seed coat and identified differentially expressed genes (DEGs) related to lipid synthesis between plants with and without hard-seededness. Our study reveals the molecular regulatory mechanisms underlying physical dormancy in alfalfa and provides a valuable genetic resource for the further hard-seededness domestication of this species.

## 2. Materials and Methods

### 2.1. Plant Material and Sample Collection

In this experiment, we used cultivated alfalfa varieties “Xinjiang Daye, WL343, and WL168” (seeds of the WL series alfalfa were obtained from Zhengdao Seed Industry Co., Ltd.,(Located in Beijing, China) and all other seeds were collected from the greenhouse) as the plant material. The alfalfa seeds were placed in a petri dish with H_2_O and then moved to a germination bag (obtained from PhenoTrait (Located in Beijing, China) with dimensions of 18 cm × 12.5 cm). After 7 d, the germinated seedlings were transferred to 1/2 Hoagland’s nutrient solution for growth and cultivation, accompanied by the replacement of a freshly prepared nutrient solution every 7 d. The plants were placed in an artificial climate incubator with a 16 h photoperiod, with day and night temperatures of 25 °C and 22 °C, respectively, and a relative humidity of 60–70%. Roots, leaves, stems, and flowers were collected from Xinjiang Daye plants. The 16–20 d seed coats of “Xinjiang Daye” and the 16–20 d developing seed coats of WL343 and WL168 were also collected. Different tissues of alfalfa were collected, immediately frozen in liquid nitrogen, and stored at −80 °C until use.

### 2.2. Seed Imbibition

The mature seeds with and without hard-seededness were soaked in a petri dish filled with water. The number of unabsorbed seeds was counted every 24 h for 7 d. The water absorption process of seeds with and without hard-seededness was observed and imaged under a stereomicroscope.

### 2.3. Transcriptome Sequencing and Analysis of Seed Coat-Specific Expression Genes

Total RNA was extracted from different tissues of alfalfa, including roots, stems, leaves, flowers, and seed coats with hard-seededness using TRIzol^®^ Reagent (Plant RNA Purification Reagent for plant tissue; Carlsbad, CA, USA), according to the manufacturer’s instructions (Invitrogen, Qingdao, China). Genomic DNA was removed with *DNase I* (TaKara, Kusatsu, Japan; Cat. No. 2270A) via incubation at 37 °C for 30 min, followed by heat inactivation at 85 °C for 5 min to eliminate the residual activity. RNA degradation and contamination were monitored on 1% agarose (Sigma-Aldrich, Cat. No. A9539) gels. Then, the RNA quality was determined using a 2100 Bioanalyser (Agilent Technologies; Santa Clara, CA, USA) and quantified using an ND-2000 (NanoDrop Technologies, Wilmington, DE, USA). Only high-quality RNA samples (OD260/280 = 1.8~2.2, OD260/230 ≥ 2.0, RNA integrity (RIN) ≥ 8.0, 28S:18S ≥ 1.0, >1 μg) were used to construct the sequencing library. There were three biological replicates for each tissue sample (each biological replicate from three independent plants grown under uniform conditions to reduce the variation).

Libraries were constructed using the Illumina NovaSeq Reagent Kit (Illumina, San Diego, CA, USA; Cat. No. 20027465): mRNA was enriched with oligo(dT) magnetic beads (Thermo Fisher Scientific, Cat. No. 61006, Waltham, MA USA) at a 1:1 (*v*/*w*) bead-to-RNA ratio and then fragmented (94 °C for 8 min, Illumina buffer Cat. No. RS-122-2101) to 200–300 bp. Double-stranded cDNA was synthesized with the SuperScript kit (Invitrogen, Cat. No. 18064014, Qingdao, China) and random hexamers (Illumina, Cat. No. RS-122-2001) (42 °C for 50 min, 70 °C for 15 min to inactivate reverse transcriptase). cDNA underwent end-repair (*T4/Klenow DNA polymerases*, NEB; 20 °C, 30 min), phosphorylation (*T4 polynucleotide kinase*, NEB; 37 °C, 30 min), and ‘A’-tailing (*Klenow 3′→5′ exo- polymerase*, NEB; 37°C, 30 min) per Illumina protocols. Target 300 bp fragments were size-selected via 2% ultra-agarose (Bio-Rad, Cat. No. 1613101), gel extraction (Qiagen, Cat. No. 28706; 30 μL elution), and then PCR amplification (15 cycles: 98 °C 10 s, 60 °C 30 s, 72 °C 30 s) with *Phusion DNA polymerase* (NEB, Cat. No. M0530).

Qualified libraries (concentration ≥ 2 nM, main peak 350–400 bp including adapters, quantified via Turner TBS380 fluorometer, Qingdao, China) were sequenced on an Illumina NovaSeq 6000 (2 × 150 bp read length, Novogene, Beijing, China) with 10 Gb clean data per sample to ensure sufficient coverage for gene expression analysis.

The raw paired-end reads were trimmed (for adapters and low-quality bases) and subjected to quality control using fastp [35] (v1.0.1; https://github.com/OpenGene/fastp, accessed on 10 October 2022), yielding high-quality clean data that guaranteed the reliability of subsequent analysis. The clean data were then aligned to the reference genome of the alfalfa cultivar Xinjiang Daye (https://figshare.com/projects/whole_genome_sequencing_and_assembly_of_Medicago_sativa/66380, accessed on 10 October 2022) using HiSat2 software [36] (v2.2.1), a tool specialized for the efficient alignment of short-read sequencing data to reference genomes. RSEM software [37] (v1.3.3; https://deweylab.biostat.wisc.edu/rsem/, accessed on 10 October 2022) was used to quantitatively analyze the expression levels of the genes. The results of the expression quantification were expressed as Transcripts per Million (TPM)—a standard normalization metric in RNA-seq that calculates the number of transcripts of a target gene per one million total transcripts of all genes in a sample, effectively correcting biases from sequencing depth and gene length [38]. This TPM normalization ensures consistent total gene expression across different samples.

Read counts of the genes obtained from RSEM quantification were analyzed using the differential analysis software DESeq2 [39] (v1.38.3; https://bioconductor.org/packages/release/bioc/html/DESeq2.html, accessed on 10 October 2022), a tool specialized for RNA-seq differential expression analysis by modeling count data with a negative binomial distribution. The selection criteria for significantly differentially expressed genes (DEGs) were defined as |log_2_(fold change, FC)| ≥ 2 and adjusted *p*-value (*p*-adjust) ≤ 0.05. The GO (Gene Ontology) and KEGG (Kyoto Encyclopedia of Genes and Genomes) databases were used to annotate and analyze the DEGs. The Goatools and KOBAS software (https://scipy.org/install/, accessed on 10 October 2022) were used to enrich and analyze the screened DEGs. The enriched DEGs were analyzed using Goatools 3.0 and KOBAS 3.0 software.

### 2.4. Differential Gene Expression Analysis and Functional Enrichment

To identify DEGs between two different samples/groups, we used the TPM method to calculate the expression level of each gene and RSEM (http://deweylab.biostat.wisc.edu/rsem/, accessed on 10 October 2022)) to quantify the gene abundances. Essentially, differential expression analysis was performed using the DESeq2, and DEGs with |log_2_ (foldchange)| ≥ 2 and *p*-adjust ≤ 0.05 were considered to be significantly different expressed genes. Functional enrichment analysis, including GO (http://www.geneontology.org, accessed on 10 October 2022) and KEGG http://www.genome.jp/kegg/, accessed on 10 October 2022), were performed to identify which DEGs were significantly enriched in GO terms and metabolic pathways at *p*-adjust ≤ 0.05 compared with the whole-transcriptome background. GO functional enrichment and KEGG pathway analysis were conducted using Goatools (https://github.com/tanghaibao/Goatools, accessed on 10 October 2022) and KOBAS.

### 2.5. Transcription Factor Family Analysis

TFs are composed of the DNA-binding domain (DBD), transactivating domain (TAD), and other TF-binding domains (Signal sensing domain (SSD)). Using the transcription factor database Plant TFDB 4.0 (Plant Transcription Factor Database, http://planttfdb.gao-lab.org/, accessed on 10 October 2022), we analyzed the information on domains contained in the transcription products of genes. Information contained in the transcription products of genes was analyzed for transcription factor prediction and family analysis by using the transcription factor database Plant TFDB 4.0 (Plant Transcription Factor Database, Version 4.0; http://planttfdb.gao-lab.org/, accessed on 10 October 2022).

### 2.6. Quantitative Real-Time PCR Analysis

To verify the expression patterns of genes observed in the RNA-seq analysis, 16 candidate genes associated with seed physical dormancy were selected for quantitative real-time PCR (qRT-PCR). The selection criteria of these genes were as follows: (1) they were specifically expressed in the alfalfa seed coat (screened from 4740 seed coat-specific genes identified in Section 3.1, with detailed information of the 4740 seed coat-specific genes provided in Supplementary Table S1); (2) they were differentially expressed between seed coats with hard-seededness (X-SC (H), 168-SC (H)) and without hard-seededness (343-SC (S), 168-SC (S)) (meeting the DEG criteria of |log_2_(foldchange)| ≥ 2 and *p*-adjust ≤ 0.05, as described in Section 2.4, and the full list of differentially expressed genes between the two types of seed coats is available in Supplementary Table S1); (3) they were involved in lipid metabolism-related pathways closely associated with seed coat impermeability (including lipid binding, lipase activity, lipid transport, and lipid metabolism pathways, as confirmed by GO/KEGG enrichment analysis in Section 3.8 and Section 3.9).

Total RNA isolation and reverse transcription with oligo (dT)18 (Invitrogen, Waltham, MA, USA) were performed as previously described. RNAs were reverse transcribed into cDNAs by using HiScript III^®^ RT SuperMix (+gDNA wiper) (Vazyme Biotech Co., Ltd., Nanjing, China). ChamQ SYBR Color qRT-PCR MasterMix (Vazyme Biotech Co., Ltd., Nanjing, China) was used for qRT-PCR, and the *MsUBC* gene was used as a reference gene. Three biological replicates were used for each sample (the biological replicates referred to independent tissue samples collected from three separate plants of the same alfalfa cultivar, ensuring consistency in genetic background and growth conditions), and each biological replicate was subjected to three technical replicates to reduce experimental errors caused by operational variability. The relative expression levels of target genes were calculated using the 2^−^ΔΔCt method (Livak method): first, the Ct values of target genes were normalized to the Ct value of the reference gene *MsUBC* to obtain ΔCt (ΔCt = Ct_target—Ct_reference); then, ΔΔCt was calculated by subtracting the ΔCt of the control group (seed coats without hard-seededness, 343-SC (S) or 168-SC (S)) from the ΔCt of the treatment group (seed coats with hard-seededness, X-SC (H) or 168-SC (H)); finally, the relative expression level was determined as 2^−^ΔΔCt.

Before calculating the relative expression levels, the following data quality control steps were performed: (1) Ct values of technical replicates were first checked for consistency—technical replicates with a Ct value variation > 0.5 were discarded to avoid operational errors; (2) for each biological replicate, the average Ct value of valid technical replicates (variation ≤ 0.5) was used for subsequent analysis; (3) outlier biological replicates were identified using the Grubbs test (α = 0.05) and excluded if they significantly deviated from the mean of the three biological replicates. Only data from valid biological and technical replicates were used to calculate the final relative expression levels and statistical significance (Student’s *t*-test, *p* < 0.05) between the treatment and control groups.

Beacon Designer 7.9 was used to design real-time quantitative primers, and the sequences of the primers used for qRT-PCR are listed in Supplementary Table S2.

### 2.7. Gene Expression Analysis of M. truncatula Homologous Genes

The homologous gene of *Ms.gene003833* in *M. truncatula* was identified by local BLAST 2.13.0(E-value ≤ 1 × 10^−5^) against the *M. truncatula* A17 genome database (https://modms.lzu.edu.cn/, accessed on 10 October 2022). Seed coats of *M. truncatula* ecotype A17 (a hard-seeded line) were collected at 5, 10, 15, 20, and 25 d after pollination (three biological replicates per time point). Total RNA extraction, cDNA synthesis, and qRT-PCR analysis were performed using the same protocols as described in Section 2.6 (Quantitative Real-Time PCR Analysis) for alfalfa samples. The relative expression levels were calculated using the 2^−ΔΔCt^ method with *MtUBQ10* as the reference gene, and statistical significance was determined by Student’s *t*-test (*p* < 0.05).

## 3. Results

### 3.1. Transcriptomic Screening of Genes Specifically Expressed in Alfalfa Seed Coat

**Figure 1:**
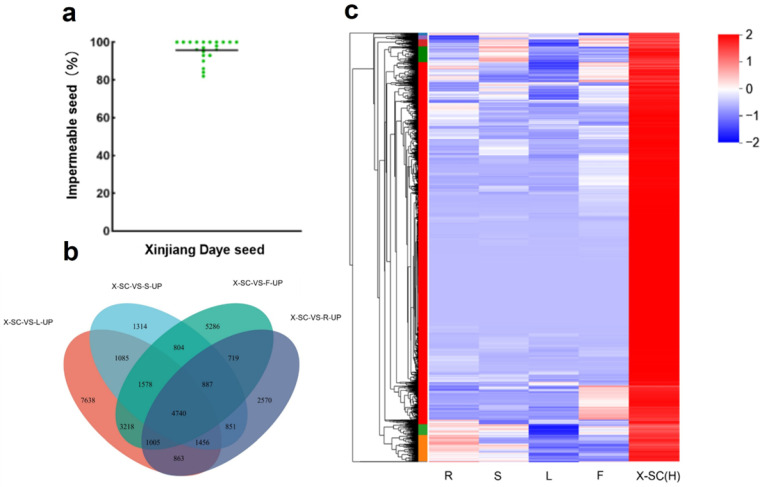
Screening the genes with higher expressions in the seed coat of Xinjiang Daye plants. (**a**) Green dots represent the impermeable seeds from each individual plant, and the horizontal line represents the overall average impermeable seed level of Xinjiang Daye. (**b**) Venn diagram analysis of differentially upregulated genes in Xinjiang Daye seed coat compared with other tissues (DEGs criteria log_2_ FC ≥ 2). (**c**) Heat map of specifically expressed genes in Xinjiang Daye seed coat (R, root; S, stem; L, leaf; F, flower; SC, seed coat).

To identify genes associated with seed physical dormancy, we screened the Xinjiang Daye plants with an average of 96% impermeable seeds (Figure 1a) and compared the gene expression pattern in different tissues, including the root, shoot, leaf, flower, and seed coat from alfalfa cultivars of Xinjiang Daye, named X-SC (H). Through Venn diagram analysis of differential expression, we identified 4740 genes specifically expressed in the seed coat (Figure 1b, Supplementary Table S1). Clustering and heat map analyses further showed that these genes were specifically expressed in the seed coat, with low or no expression in other tissues (Figure 1b,c).

### 3.2. Gene Ontology Annotation and Enrichment of Genes Specifically Expressed in Seed Coat

**Figure 2:**
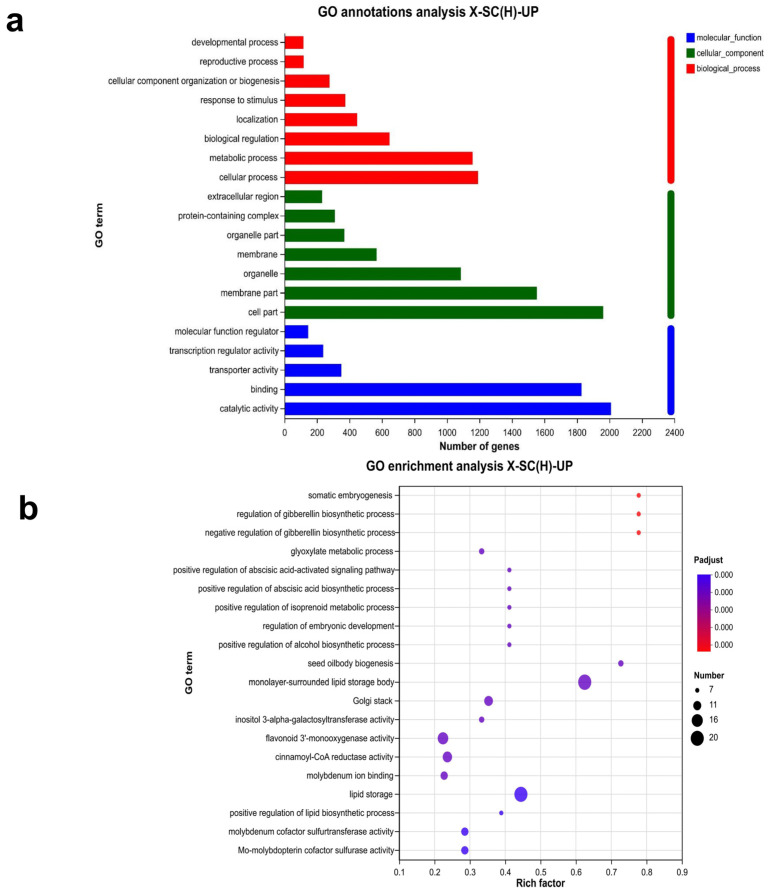
GO annotations and enrichment analysis of specifically expressed genes in seed coats. (**a**) GO annotations of specifically expressed genes in seed coats. (**b**) Enrichment analysis of specifically expressed genes in seed coats.

The 4740 genes specifically expressed in the seed coat were subjected to gene ontology (GO) analysis. This analysis classified them into three main categories: biological processes, cellular components, and molecular functions (Figure 2a). These genes were mainly enriched in the monolayer-surrounded lipid storage bodies (20 genes) and lipid storage (20 genes) pathways (Figure 2b). A directed acyclic graph analysis revealed that lipid storage affected cellular composition, leading us to hypothesize that the formation of hard-seededness was related to the lipid content (Supplementary Figure S1). In order to identify genes enriched in the cell wall and lipid pathways, the top 10 enriched GO terms were selected to generate an enrichment chord diagram (Supplementary Figure S2). The results demonstrated that 23 genes in the cell wall-related pathway were primarily enriched in seed biogenesis, positive regulation of the alcohol biosynthetic process, and positive regulation of isoprenoids (Supplementary Figure S2). The lipid-associated pathway contained 179 genes, mainly involved in lipid storage, catabolism, and binding (Supplementary Figure S3).

### 3.3. Kyoto Encyclopedia of Genes and Genomes Annotation and Enrichment of Genes Specifically Expressed in Seed Coat

Kyoto Encyclopedia of Genes and Genomes (KEGG) annotation of the gene set specifically expressed in the seed coat classified the 4740 genes into five main categories: metabolism, environmental information processing, genetic information processing, cellular processes, and organismal systems (Supplementary Figure S4a). The results showed that most genes were enriched in carbohydrate metabolism, followed by lipid, amino acid, and other secondary metabolic pathways. Enrichment analysis showed that the genes were mainly enriched in the phenylpropane biosynthesis and flavonoid biosynthesis pathways (Supplementary Figure S4b).

### 3.4. Transcription Factor Prediction of Genes Specifically Expressed in Seed Coat

**Figure 3:**
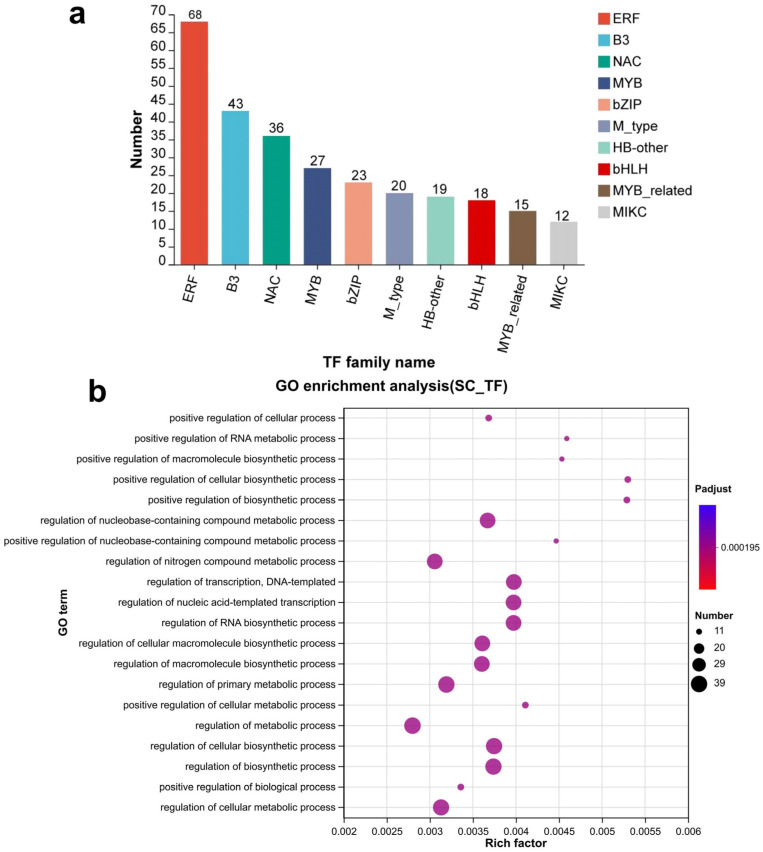
Gene transcription factor prediction among seed coat-specific expression genes. (**a**) The statistics of seed coat-specific expressed transcription factor family. (**b**) GO enrichment analysis of seed coat-specific expressed transcription factor genes.

Twenty-six transcription factor families were predicted to be associated with the 4740 seed coat-specific genes (Figure 3a). The three largest families were ERF (68), B3 (43), and NAC (36). Other families included (27), bZIP (23), M_type (20), HB-other (19), bHLH (18), MYB-related (15), and MIKC (12). GO enrichment analysis revealed that the transcription factors were mainly involved in the regulation of metabolism, cellular biosynthesis, transcription, and other processes (Figure 3a).

### 3.5. Hard-Seededness Variation in Different Alfalfa Cultivars

In this study, three alfalfa cultivars were tested to analyze the hard-seededness variation, including two commercial cultivars (“WL343” and “WL168”) and one local cultivar (“Xinjiang Daye”) (detailed in Section 2.1). To study the hard-seededness variation between different alfalfa cultivars, we placed alfalfa seeds from different cultivars in water for 7 d and then, at 24 h intervals, counted the number of seeds that did not absorb water. The number of non-absorbing seeds gradually decreased as the time in water increased, and after 7 d, there were very few non-absorbing seeds in WL168. After 7 d, the hard-seededness rate of WL168 was 5% (Supplementary Figure S5a) and that of WL343 was 15% (Supplementary Figure S5b). By contrast, even with increased soaking time, the seeds with physical dormancy did not soften or swell (Supplementary Figure S5c,d).

### 3.6. Phenotypic Changes of Seeds with and Without Physical Dormancy

**Figure 4:**
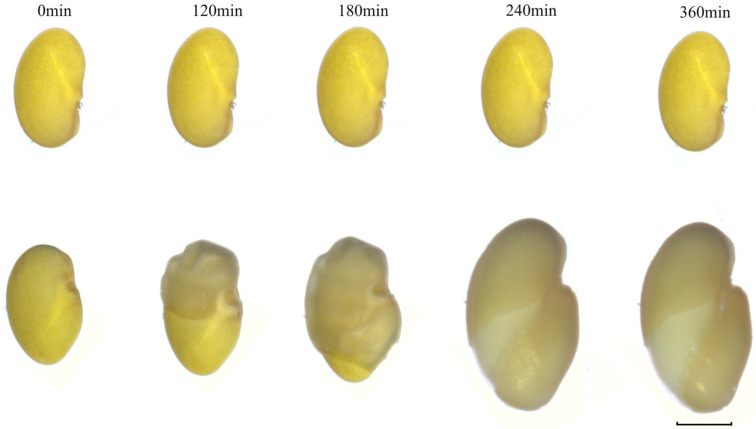
Imbibition of seeds with physical dormancy (**top row**) and without physical dormancy (**bottom row**) in sterile water. (Scale bar, 2 mm).

We determined the changes in water uptake for seeds with and without physical dormancy. The seeds were placed in distilled water. After 120 min, the seeds started to absorb water, and the seed coat became wrinkled. At 240 min, the seeds gradually swelled, and the seed coat became smooth and cracked. With the increase in the imbibition time, the seed cracks became more prominent, and the radicle started growing. Monitoring the absorption process in 20 seeds without physical dormancy, we noticed that the absorbing seeds always started suckering water at a point near the end of the seed radicle, and then their seed coats gradually wrinkled, ultimately causing the seeds to absorb water and sprout. In contrast, with increasing hard-seededness, the seeds did not show any signs of water absorption or sprouting (Figure 4).

### 3.7. Transcription Analysis of Seed Coats from Seeds with and Without Physical Dormancy

**Figure 5:**
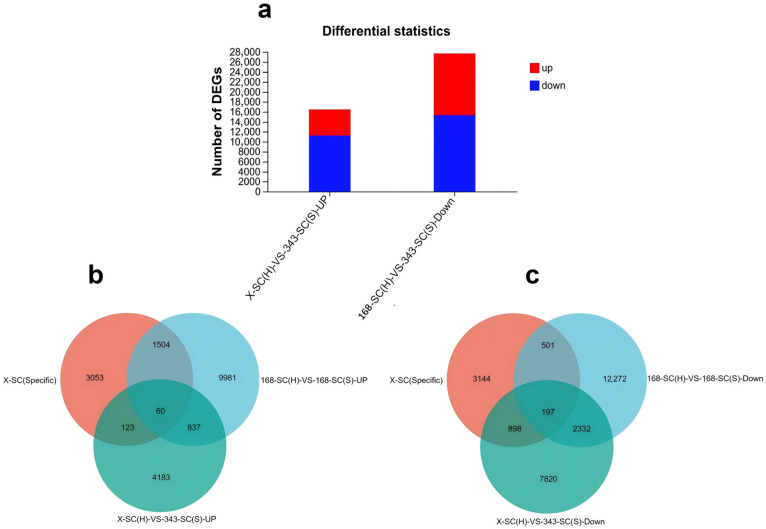
Differential expression analysis of genes related to seed physical dormancy. (**a**) Statistics of differentially expressed genes in seed coats with and without physical dormancy. Red represents upregulation, and blue represents downregulation. Venn diagrams of the differentially expressed upregulated (**b**) and downregulated (c) DEGs shared by 168-SC (S)-VS-168-SC (H), 343-SC (S)-VS-X-SC (H), and X-SC (Specific) (DEGs criteria log_2_ FC ≥ 2).

Alfalfa seeds coats, X-SC (H) and 168-SC (H) with hard-seededness and 343-SC (S) and 168-SC (S) without hard-seededness, were subjected to transcriptome analysis. In the 343-SC (S) and X-SC (H) samples, 5203 genes were significantly upregulated, while 11,247 genes were significantly downregulated (Figure 5a). A comparison between the 168-SC (H) and 168-SC (S) samples revealed 12,382 significantly upregulated DEGs and 15,302 downregulated DEGs (Figure 5a). By analyzing the Venn diagrams of the DEGs shared between seed coats with and without physical dormancy and specifically expressed within seed coats compared with other plant tissue, we identified 60 up- and 197 downregulated genes, named SC-UP and SC-DOWN, respectively (Figure 5b,c, Supplementary Table S1).

### 3.8. GO Annotation and Enrichment Analysis of DEGs in Seed Coats with and Without Physical Dormancy

**Figure 6:**
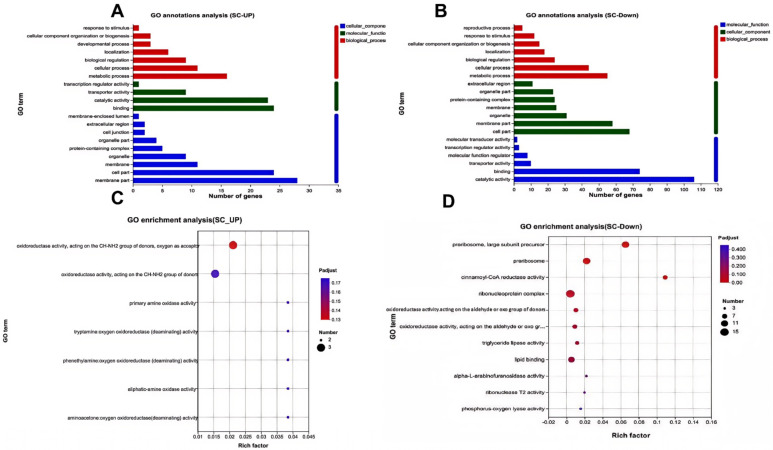
GO annotation and enrichment analysis of SC-UP and SC-DOWN differentially expressed genes. (**a**) GO annotation of differentially upregulated genes in SC-UP. (**b**) GO annotation of differentially downregulated genes in SC-DOWN. (**c**) GO enrichment analysis of differentially upregulated genes in SC-UP. (**d**) GO enrichment analysis of differentially downregulated genes in SC-DOWN.

GO analysis showed that SC-UP and SC-DOWN expression differed among biological processes, cellular components, and molecular functions (Figure 6a,b). SC-UP were mainly enriched in oxidoreductase activity (Figure 6c), and SC-DOWN were enriched in lipid binding and triglyceride lipase activity (Figure 6d), both of which are associated with seed coat formation. The GO-directed acyclic plot of SC-DOWN downregulated genes showed a significant correlation between the seed coat with physical dormancy and lipid binding pathway, suggesting that a high lipid content may contribute to seed coats with hard-seededness (Supplementary Figure S6).

### 3.9. KEGG Annotation and Enrichment of DEGs in Hard and Non-Hard Seed Coats

The KEGG pathways of SC-DOWN were involved in five categories: metabolism, environmental information processing, genetic information processing, cellular processes, and biological systems (Supplementary Figure S7a). Among them, lipid metabolism pathways showed significant gene expression differences throughout the process. KEGG enrichment analysis of SC-DOWN showed that more DEGs were in the phenylpropane biosynthesis and flavonoid biosynthesis pathways (Supplementary Figure S7b. The KEGG pathways of SC-UP were involved in five main categories: metabolic pathways, metabolism, environmental information processing, genetic information processing, and biological systems (Supplementary Figure S7c). Sixty SC-UP DEGs were mainly enriched in KEGG pathways such as ABC transporters, tropane, piperidine and pyridine alkaloid biosynthesis, isoquinoline alkaloid biosynthesis, and the tyrosine metabolism pathway (Supplementary Figure S7d).

### 3.10. Screening Genes Associated with Seeds’ Physical Dormancy

The seeds’ physical dormancy is associated with cell wall and lipid synthesis, and genes involved in the synthesis of cuticle layer have been identified to control seed hardness formation. We conducted a screening to identify genes that regulate the formation of hard-seededness through their involvement in cuticle layer biosynthesis. Through GO term and KEGG pathway enrichment analyses of DEGs in seed coats, four pathways related to lipid and cell wall synthesis were determined, which were lipid binding, lipase activity, lipid transport, and lipid metabolism. From these pathways, sixteen genes were identified, including eight genes in the lipid binding pathway, five genes in the lipase activity pathway, one gene in the lipid transport pathway, and two genes in the lipid metabolism pathway (Table 1). These genes are specifically expressed in the seed coats and compared with the seed coats without hard-seededness, these genes are significantly upregulated in the seed coats with hard-seededness (Supplementary Figure S8a–c).

### 3.11. Quantitative Real-Time PCR Verification of Screened DEGs in Seed Coats with Hard-Seededness

Sixteen genes that are specifically expressed in the seed coats and related to hard-seededness were selected for quantitative real-time PCR (qRT-PCR) to confirm the expression patterns observed in the RNA sequencing (RNA-seq) analysis. The qRT-PCR results indicated that 13 out of 16 genes were specifically expressed in seed coats and had low or no expression in other tissues (Supplementary Figure S9), which was consistent with the transcriptome analysis. Among the thirteen seed coat-specific expressed genes, seven exhibited higher expression level in seed coats with hard-seededness, compared with seed coats without hard-seededness, which support the results from the transcriptome analysis. However, there were several other genes that did not align with the transcriptome results. These genes were either not specifically expressed in the seed coats or had higher expression level in seed coats without hard-seededness (Supplementary Figure S10).

### 3.12. BLAST and Bioinformatics Analysis of Candidate Genes Associated with Hard-Seededness

**Figure 7:**
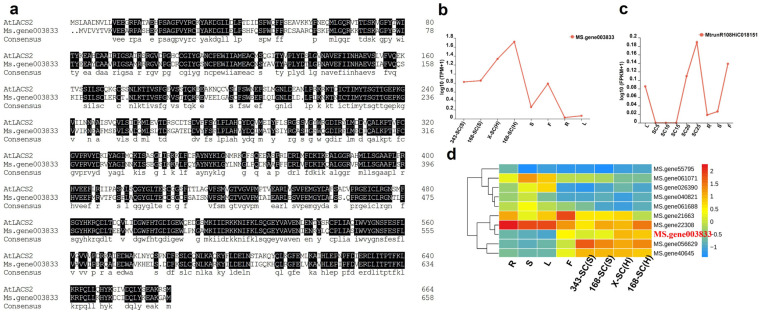
Bioinformatics analysis of candidate gene *Ms.gene003833*. (**a**) Alignment of *Ms.gene003833* gene with *AtLACS2*. (**b**) The expression of *Ms.gene003833* in different tissues. (**c**) The expression of *MtrunR108HiC018151* in different tissues. (**d**) A gene clustering heat map of *Ms.gene003833* gene family.

The 16 candidate genes associated with hard-seededness were uploaded to NCBI for online BLAST analysis, and it was found that the *Ms.gene003833* gene was highly similar to the *LACS2* (Long-Chain Acyl-CoA Synthetase 2) gene of the *LACs* family in *Arabidopsis thaliana*, with a similarity of 67% (Figure 7a). The *Ms.gene003833* gene was not only specifically expressed in the seed coat but also had higher expression level in seed coats with hard-seededness, compared with seed coats without hard-seededness (Figure 7b). A homologous gene of *Ms.gene003833* (*MtrunR108HiC018151*) was identified in *M. truncatula* ecotype A17 (a hard-seeded line) via local BLAST against the *M. truncatula* genome database (https://modms.lzu.edu.cn/, accessed on 10 October 2022). The expression profile of this gene during seed coat development (5–25 d after pollination) was generated in this study, with detailed experimental procedures included in Section 2.7 (Gene expression analysis of *M. truncatula* homologous genes). Briefly, its expression was significantly higher than that in the other tissues (Figure 7c). By conducting BLAST in the alfalfa genome, there were 10 genes in its gene family. Cluster analysis of this gene family in different tissues revealed that three genes were specifically expressed in the seed coat, but only the *Ms.gene003833* gene had a higher expression level in the seed coat with hard-seededness compared with seed coats without hard-seededness (Figure 7d).

### 3.13. Analysis of Homologous Genes of MtKCS12, MtKNOX4, GmHs1-1, and GmqHs1 in Alfalfa

**Figure 8:**
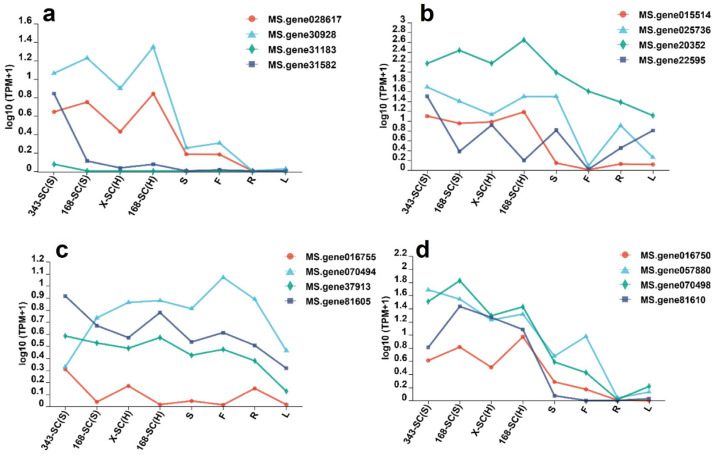
The homologous genes’ expression levels of hard-seededness-related genes *MtKCS12* (**a**), *MtKNOX4* (**b**), *MsHs1-1* (**c**), and *MsqHS1* (**d**) in different tissues of alfalfa.

We utilized the genes *MtKCS12* and *MtKNOX4*, which were reported in *M. truncatula* for the regulation of hard-seededness, to perform a local BLAST analysis of the alfalfa genome. Our analysis revealed the presence of four homologs of *MtKCS12*, three of which were specifically expressed in the seed coats, namely *Ms.gene028617*, *Ms.gene30928*, and *Ms.gene31582*. In contrast, the expression level of *Ms.gene 31183* is very low in all plant tissues, including seed coats, roots, stems, leaves, and flowers (Figure 8a). Among the candidate genes, the *Ms.gene 31183* exhibits exclusive seed coat-specific expression, as it was detected only in seed coat tissues. By comparison, the *Ms.gene 028617* shows a similar seed coat-preferential expression pattern, characterized by trace expression in roots and predominant expression in seed coats (Figure 8a).

Similarly, we discovered four homologous genes of *MtKNOX4*, which were specifically expressed in the seed coats, and there were no significant expression differences in seed coats with and without hard-seededness (Figure 8b). We also performed a homology gene search in the alfalfa genome for the genes *GmHs1-1* and *GmqHs1* in soybean. The results showed *GmHs1-1* exhibited homology with *Ms.gene81605*, *Ms.gene070494*, *Ms.gene37913*, and *Ms.gene016755*. *GmqHs1* demonstrated homology with *Ms.gene016750*, *Ms.gene81610*, *Ms.gene070498*, and *Ms.gene057880*. Among the four homologs of *GmHs1-1* in alfalfa, there were no significant differences in expression observed between the seed coats and other plant tissues (Figure 8c). On the other hand, the homologs of *GmqHs1* were specifically expressed in the seed coats; however, there were no differences in expression between seed coats with and without hard-seededness (Figure 8d).

## 4. Discussion

The presence of physical dormancy in seeds can cause various challenges in agricultural production, especially in legume forage production, such as reduced germination rates, uneven seedling emergence, difficulty in establishment, increased seed usage, and increased agricultural costs. One crucial characteristic during crop domestication is the reduction in or elimination of seed dormancy, which enables uniform and timely seed germination [34].

The composition of carbohydrates, hydroxylated fatty acids, or phenol compounds in seed coats may be related to the level of seed coat permeability. The morphological structure, phenolic content, and cuticle composition of legume seeds have been investigated to answer the mystery of physical dormancy [24,40,41]. Until recent years, genes directly impacting the physical dormancy of seeds were identified based on map-based cloning in soybean, including *GmHs1-1* [22], which encodes a transmembrane protein associated with seed coat impermeability and calcium content. Similarly, *GmqHs1* encoding an endo-1,4-β-glucanase, affects hard-seededness when its polymorphisms modify the enzyme’s substrate affinity. In the model legume *M. truncatula*, the knockout of the *KNOX4* gene results in dysfunctional palisade cuticles and increased water permeability of seeds [27]. The downstream *KCS12* gene, regulated by KNOX4, plays a vital role in the production of very long-chain fatty acids (VLCFA) and, consequently, affects seed physical dormancy [27].

Alfalfa, known as the “queen of forage”, is a perennial legume used as forage grass that is a cross-pollinated, tetraploid, and dicotyledonous plant [42]. Alfalfa can develop seeds with hard-seededness at a rate of 20–80% in different varieties [31], which complicates sowing and affects production efficiency. Currently, the molecular mechanism behind seed physical dormancy in alfalfa remains unclear, as each alfalfa individual has its own recombination history, making each individual a unique genotype. Traditional genetic approaches, such as forward genetics and map-based cloning, are challenging due to the complex genomic information and self-incompatibility of alfalfa. However, RNA-seq has been successfully utilized in the study of gene screening changes in legume plants such as *M. truncatula* [43], alfalfa [44], soybean [45], faba bean [46], and chickpea [47].

In this study, we performed RNA-seq analysis and identified 4,740 genes specifically expressed in the alfalfa seed coat. GO enrichment analysis of these seed coat-specific genes revealed significant enrichment in the lipid storage pathway. Enrichment of cell wall- and lipid-related pathways has previously been associated with changes in physical seed dormancy [27,48]. In our study, genes related to lipid binding and triglyceride lipase activity were downregulated in hard-seeded lines, whereas genes involved in oxidoreductase activity were upregulated. This differential expression supports the hypothesis that lipid content in the seed coat may serve as a key factor in physical dormancy, consistent with findings in other legumes such as *M. truncatula* and *G. max*. The further detailed analysis of RNA-seq in alfalfa seed coats with and without hard-seededness has pinpointed a specific set of genes that exhibit differential expression. Our finding revealed that 60 genes were differentially upregulated, and 197 genes were differentially downregulated in seed coats with hard-seededness compared with seed coats without hard-seededness (Figure 5b,c). GO enrichment analysis showed that the upregulated genes were mainly enriched in the oxidoreductase activity pathway, and the downregulated genes were enriched in the lipid binding and triglyceride lipase activity pathways (Figure 6). This further confirms that the seed coat with hard-seededness is significantly associated with the lipid binding pathway and that lipid content may contribute to hard-seededness. One of screened genes primarily linked to lipid synthesis pathways, *Ms.gene003833*, exhibited higher expression levels in seed coats with hard-seededness, suggesting its potential role in determining seed physical dormancy. Its homologous gene in Arabidopsis, *LACS2*, is known for its involvement in cuticle or cuticle wax synthesis, with mutants displaying reduced seed yield and germination rates [49,50]. Our analyses also indicate that the expression of its homologous gene in *M. truncatula* is concomitant with seed coat development, correlating with an increase in hard-seededness development in seed.

Overall, in this study, we identified numerous genes involved in hard-seededness. Notably, we successfully detected homologs of *GmHs1-1*, *GmqHs1*, *MtKCS12*, and *MtKNOX4*—genes previously characterized as key regulators of physical dormancy in other legumes—and confirmed their differential expression between hard-seeded and non-hard-seeded alfalfa lines. This research advances our understanding of the molecular mechanisms underlying physical dormancy in legume seeds and highlights the complex genetic and biochemical processes that contribute to this trait. By linking specific genes to seed coat development and dormancy, we provide new avenues for improving seed quality and germination rates in alfalfa and other leguminous crops. Our findings offer novel insights into the relationship between seed coat-specific gene expression and physical dormancy, making a significant contribution to the understanding of this phenomenon in legume crops—especially alfalfa—and providing valuable genetic resources for further exploration and potential applications in agricultural practices.

## 5. Conclusions

This study aimed to investigate the genetic factors contributing to physical dormancy in alfalfa seeds by examining transcriptomic differences associated with hard-seededness. Hard-seededness is an important trait that restricts seed water absorption and germination in alfalfa. In this study, we successfully identified a series of genes that are highly expressed in the alfalfa seed coat and found that these genes are enriched in the lipid metabolism pathway. Several of these genes showed differential expression levels between seeds with and without hard-seededness. Verification by qRT-PCR analysis revealed that 7 out of 16 genes have the potential to control physical dormancy in alfalfa seeds. These findings enhance our understanding of the molecular mechanisms underlying physical seed dormancy in alfalfa. Further research is needed to validate the functional roles of the identified genes and to establish their connection to the hard-seededness phenotype. Overall, our study provides valuable genetic resources and new insights for future breeding in forage crops.

## Figures and Tables

**Table 1:** DEGs specifically upregulated in seed coats with hard-seededness, compared with seed coats without hard-seededness.

Gene ID	Pathway	Non-Redundant Protein Sequence Database
*MS.gene071857*	lipid binding	major allergen Pru ar 1-like protein [Trifolium pratense]
*MS.gene04019*	lipid binding	pleckstrin homology domain-containing protein 1 [Medicago truncatula]
*MS.gene72825*	lipid binding	acyl-CoA-binding protein [Medicago truncatula]
*MS.gene87096*	lipid binding	non-specific lipid-transfer protein 1 [Medicago truncatula]
*MS.gene27472*	lipid binding	lipid transfer protein [Medicago truncatula]
*MS.gene27474*	lipid binding	lipid transfer protein [Medicago truncatula]
*MS.gene00236*	lipid binding	major allergen Pru ar 1-like protein [Trifolium pratense]
*MS.gene89266*	lipid binding	putative lipid-transfer protein DIR1 isoform X1 [Medicago truncatula]
*MS.gene27869*	lipase activity	GDSL esterase/lipase At5g45670 [Medicago truncatula]
*MS.gene019909*	lipase activity	triacylglycerol lipase 2 [Medicago truncatula]
*MS.gene36001*	lipase activity	triacylglycerol lipase 2 [Medicago truncatula]
*MS.gene74700*	lipase activity	triacylglycerol lipase 2 [Medicago truncatula]
*MS.gene72397*	lipase activity	triacylglycerol lipase-like protein [Medicago truncatula]
*MS.gene003833*	lipid metabolism	long chain acyl-CoA synthetase 2 [Medicago truncatula]
*MS.gene003796*	lipid metabolism	probable 1-acyl-sn-glycerol-3-phosphate acyltransferase 5 [Medicago truncatula]
*MS.gene87717*	lipid transport	non-specific lipid-transfer protein 2 [Medicago truncatula]

## Data Availability

The original contributions presented in this study are included in the article/Supplementary Material. Further inquiries can be directed to the corresponding author.

## Supplementary Materials

The following supporting information can be downloaded at https://www.mdpi.com/article/10.3390/genes16121438/s1, Figure S1: GO directed acyclic graph analysis of 4740 seed coat-specific expression genes; Figure S2: Enrichment chords of cell wall pathway; Figure S3: Enrichment chords of lipid pathway; Figure S4: KEGG annotations and enrichment analysis of specifically expressed genes in seed coats; Figure S5: Percentage of impermeable (hard) seeds in water at multiple points; Figure S6: GO directed acyclic plot analysis of non-hard seed coat differential expression downregulated genes compared to hard seed coat; Figure S7: KEGG annotation and enrichment analysis of differentially expressed genes in hard and non-hard seed coats; Figure S8: Expression of genes involved in the formation of hardseededness in seed coats and other tissues; Figure S9: Validation of the expression levels of Seed coat-specific genes by qRT-PCR; Figure S10: Quantification of the expression levels of genes involved in the formation of hard seed and non-hard seed coats using qRT-PCR; Table S1: 4740 X-SC(specific), 60 SC-UP and 197 SC-Down genes are obtained using the Venn diagram. Table S2. Sequences of primers used in qRT-PCR.
