# Supplementary material for: Global Transcriptome Analysis Reveals the Molecular Mechanism Underlying Seed Physical Dormancy Formation in Medicago sativa
Source: Genes (Basel). 2025 Dec 1;16(12):1438. doi: 10.3390/genes16121438 (PMC12732859; doi:10.3390/genes16121438)
Supplement: Supplementary file 1 [file genes-16-01438-s001.zip › Supplementary Figures 24 02.16.pptx]

## Slide 1
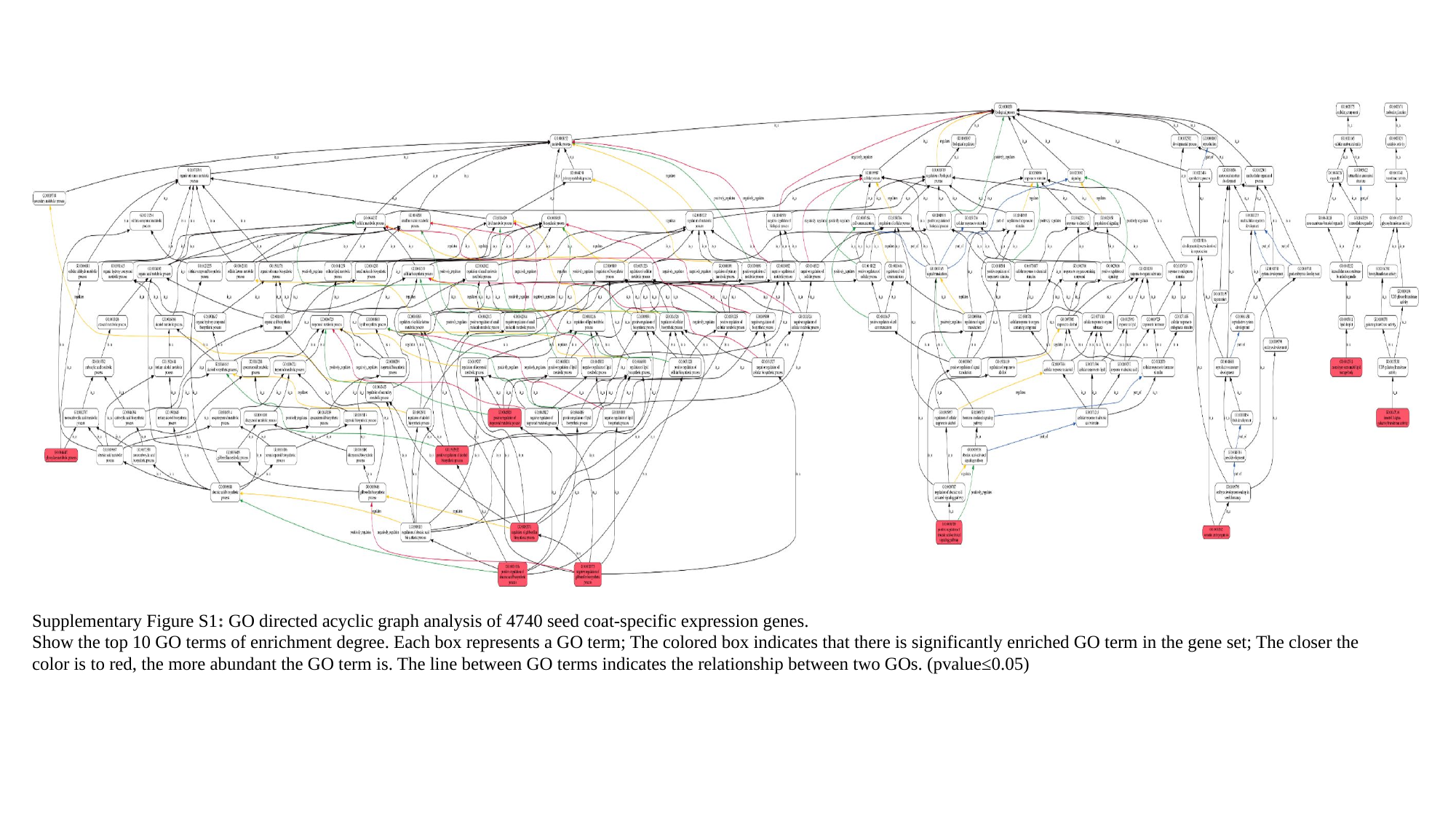

Supplementary Figure S1: GO directed acyclic graph analysis of 4740 seed coat-specific expression genes.
Show the top 10 GO terms of enrichment degree. Each box represents a GO term; The colored box indicates that there is significantly enriched GO term in the gene set; The closer the color is to red, the more abundant the GO term is. The line between GO terms indicates the relationship between two GOs. (pvalue≤0.05)

## Slide 2
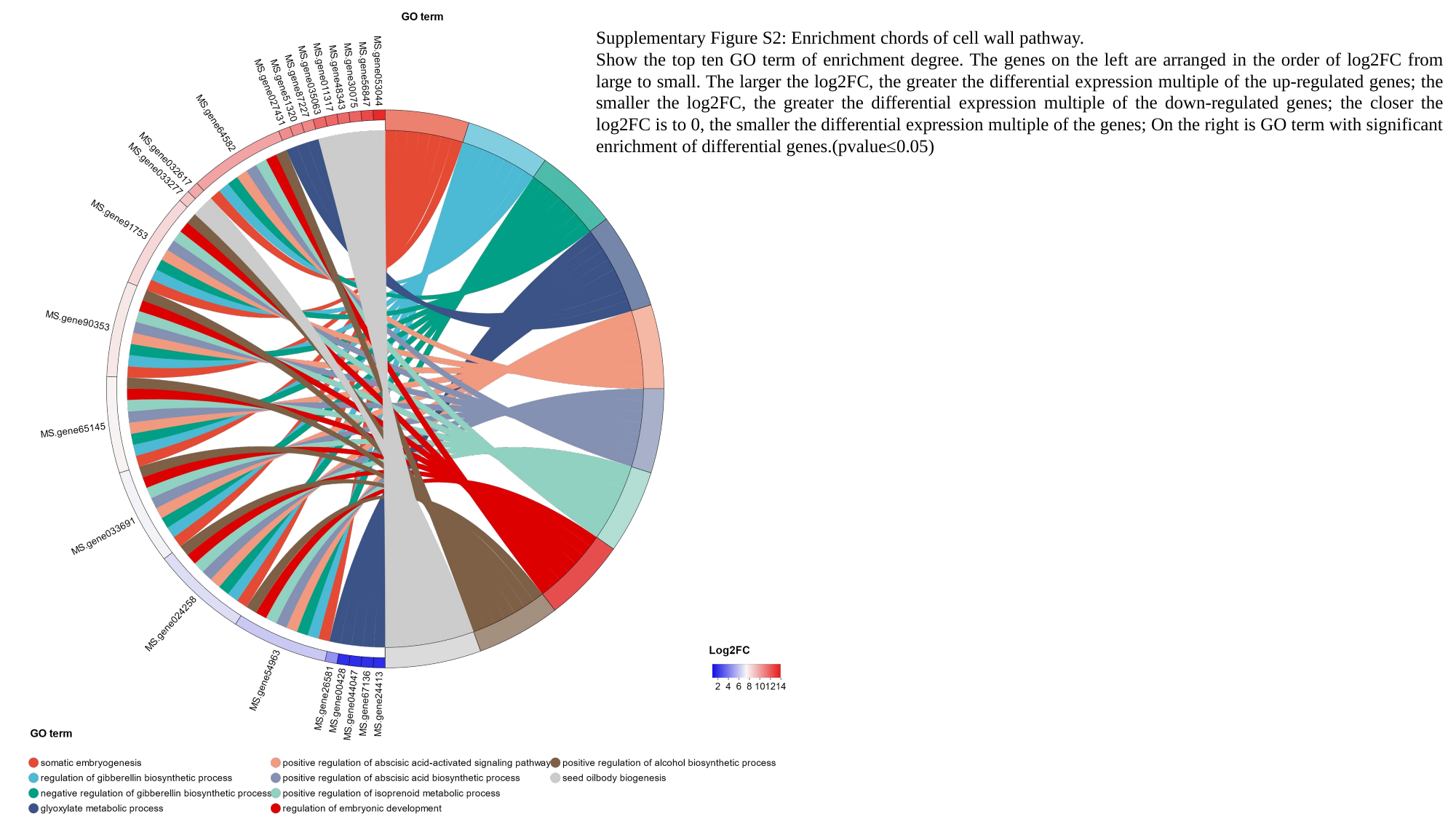

Supplementary Figure S2: Enrichment chords of cell wall pathway.
Show the top ten GO term of enrichment degree. The genes on the left are arranged in the order of log2FC from large to small. The larger the log2FC, the greater the differential expression multiple of the up-regulated genes; the smaller the log2FC, the greater the differential expression multiple of the down-regulated genes; the closer the log2FC is to 0, the smaller the differential expression multiple of the genes; On the right is GO term with significant enrichment of differential genes.(pvalue≤0.05)

## Slide 3
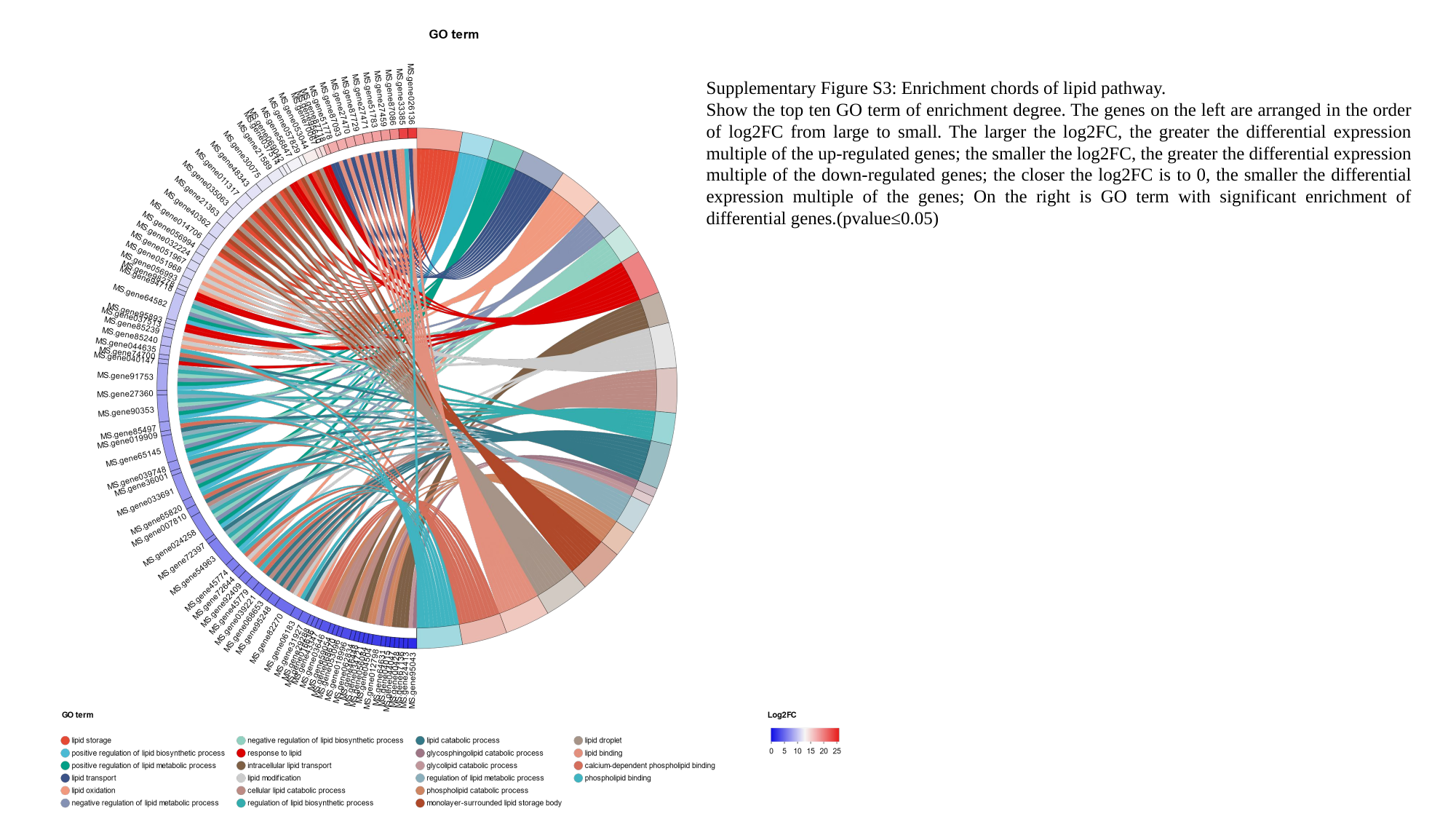

Supplementary Figure S3: Enrichment chords of lipid pathway.
Show the top ten GO term of enrichment degree. The genes on the left are arranged in the order of log2FC from large to small. The larger the log2FC, the greater the differential expression multiple of the up-regulated genes; the smaller the log2FC, the greater the differential expression multiple of the down-regulated genes; the closer the log2FC is to 0, the smaller the differential expression multiple of the genes; On the right is GO term with significant enrichment of differential genes.(pvalue≤0.05)

## Slide 4
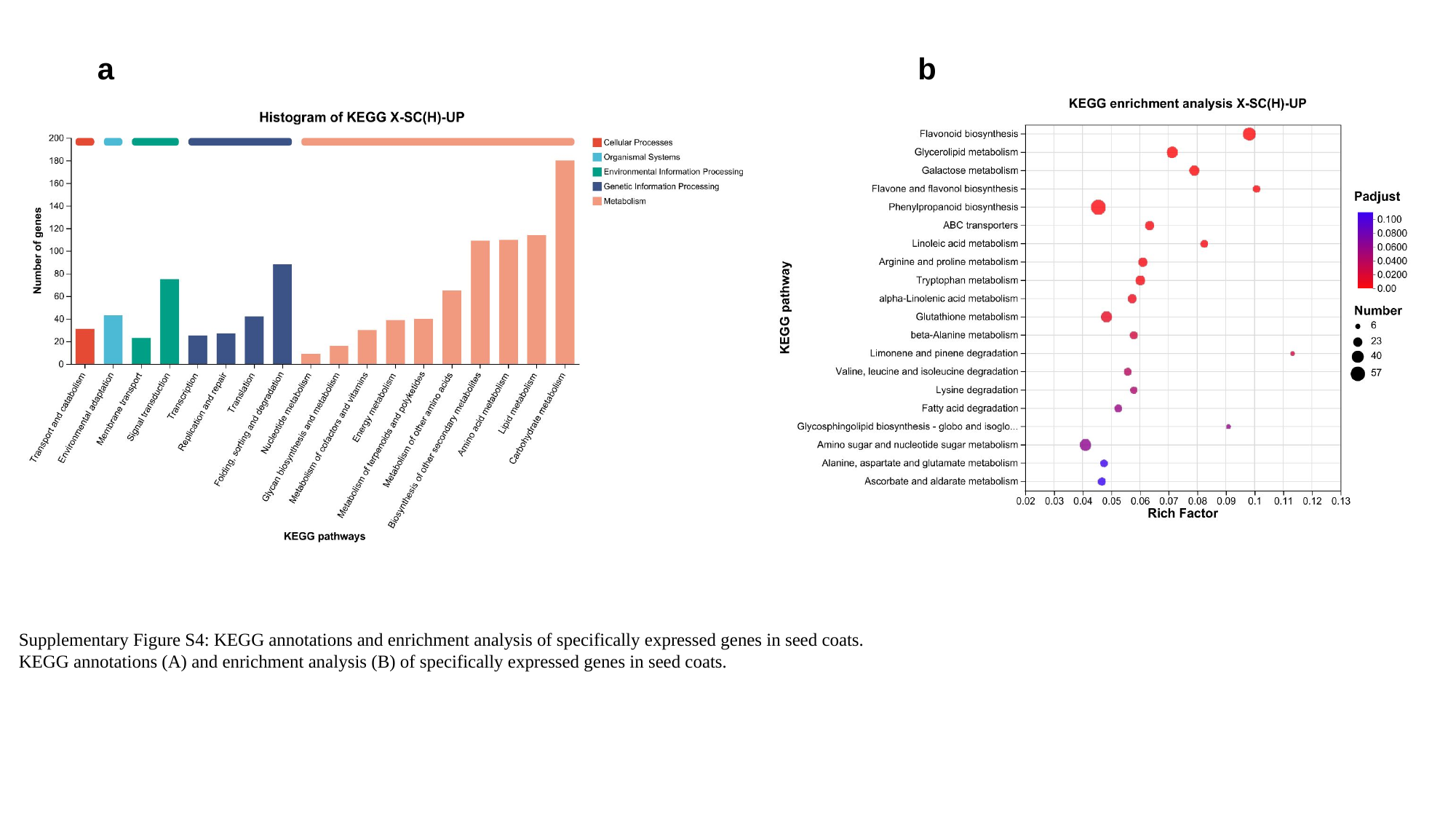

a
b
Supplementary Figure S4: KEGG annotations and enrichment analysis of specifically expressed genes in seed coats.
KEGG annotations (A) and enrichment analysis (B) of specifically expressed genes in seed coats.

## Slide 5
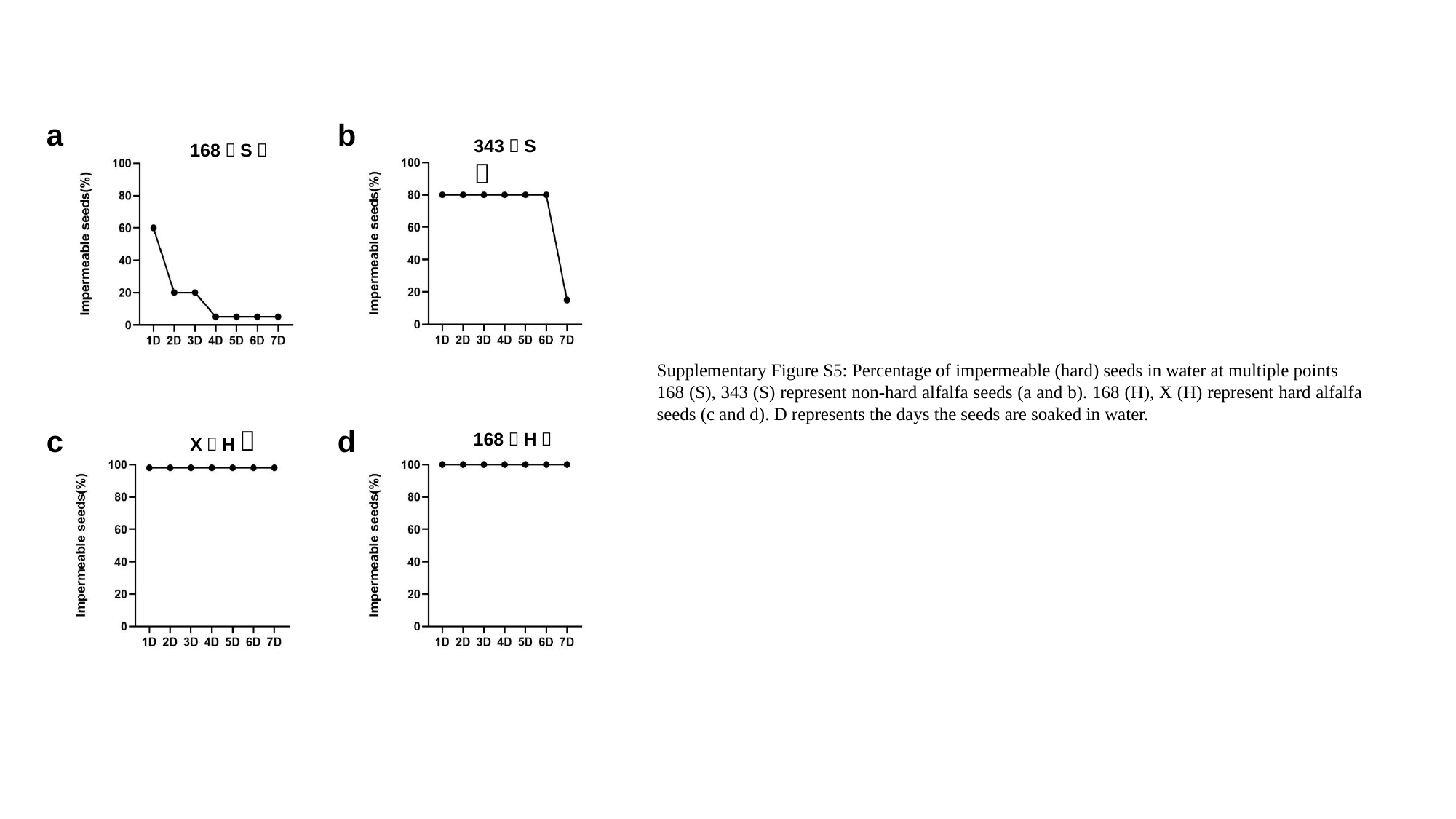

a
b
343（S）
168（S）
X（H）
168（H）
c
d
Supplementary Figure S5: Percentage of impermeable (hard) seeds in water at multiple points
168 (S), 343 (S) represent non-hard alfalfa seeds (a and b). 168 (H), X (H) represent hard alfalfa seeds (c and d). D represents the days the seeds are soaked in water.

## Slide 6
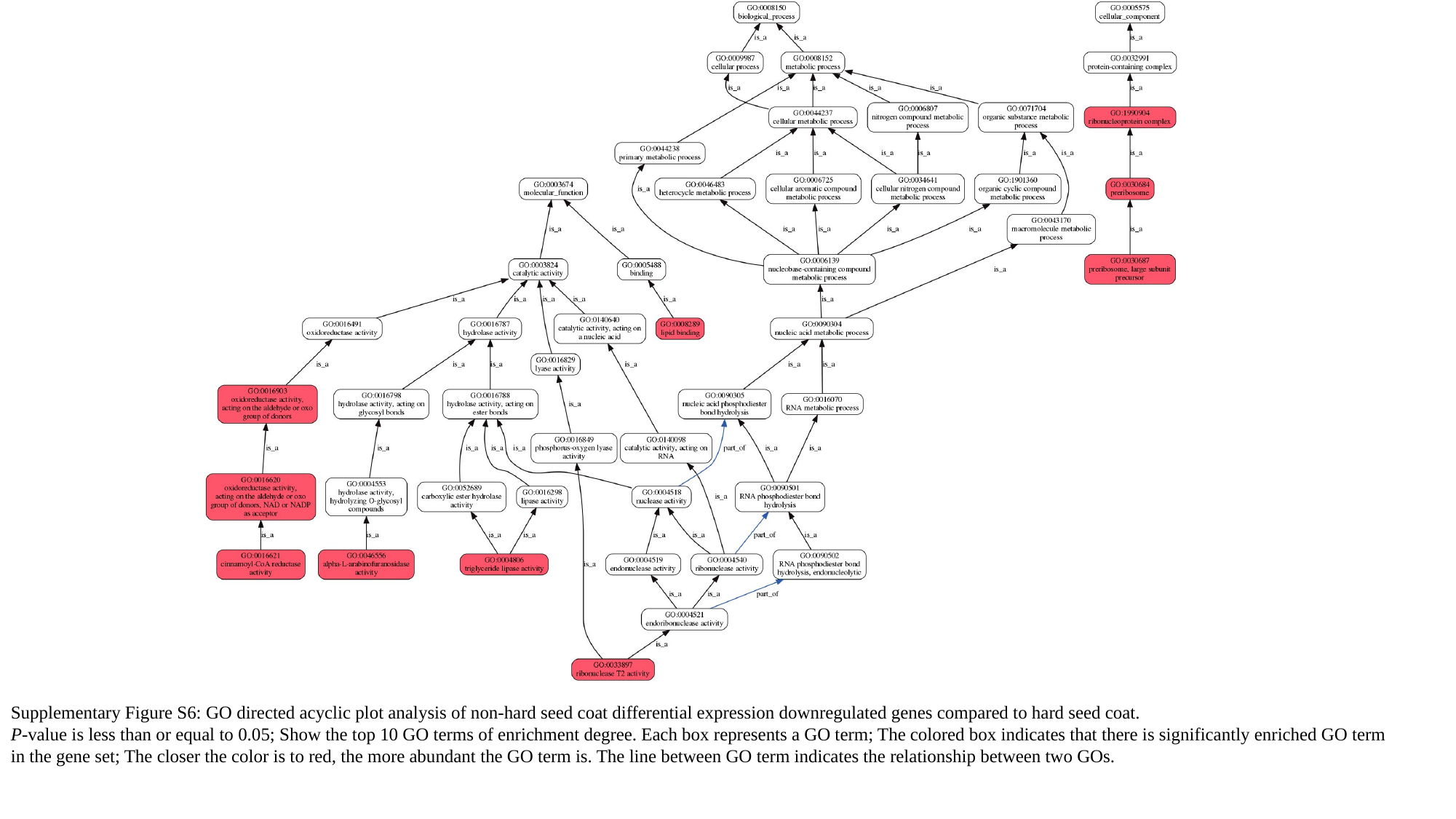

Supplementary Figure S6: GO directed acyclic plot analysis of non-hard seed coat differential expression downregulated genes compared to hard seed coat.
P-value is less than or equal to 0.05; Show the top 10 GO terms of enrichment degree. Each box represents a GO term; The colored box indicates that there is significantly enriched GO term in the gene set; The closer the color is to red, the more abundant the GO term is. The line between GO term indicates the relationship between two GOs.

## Slide 7
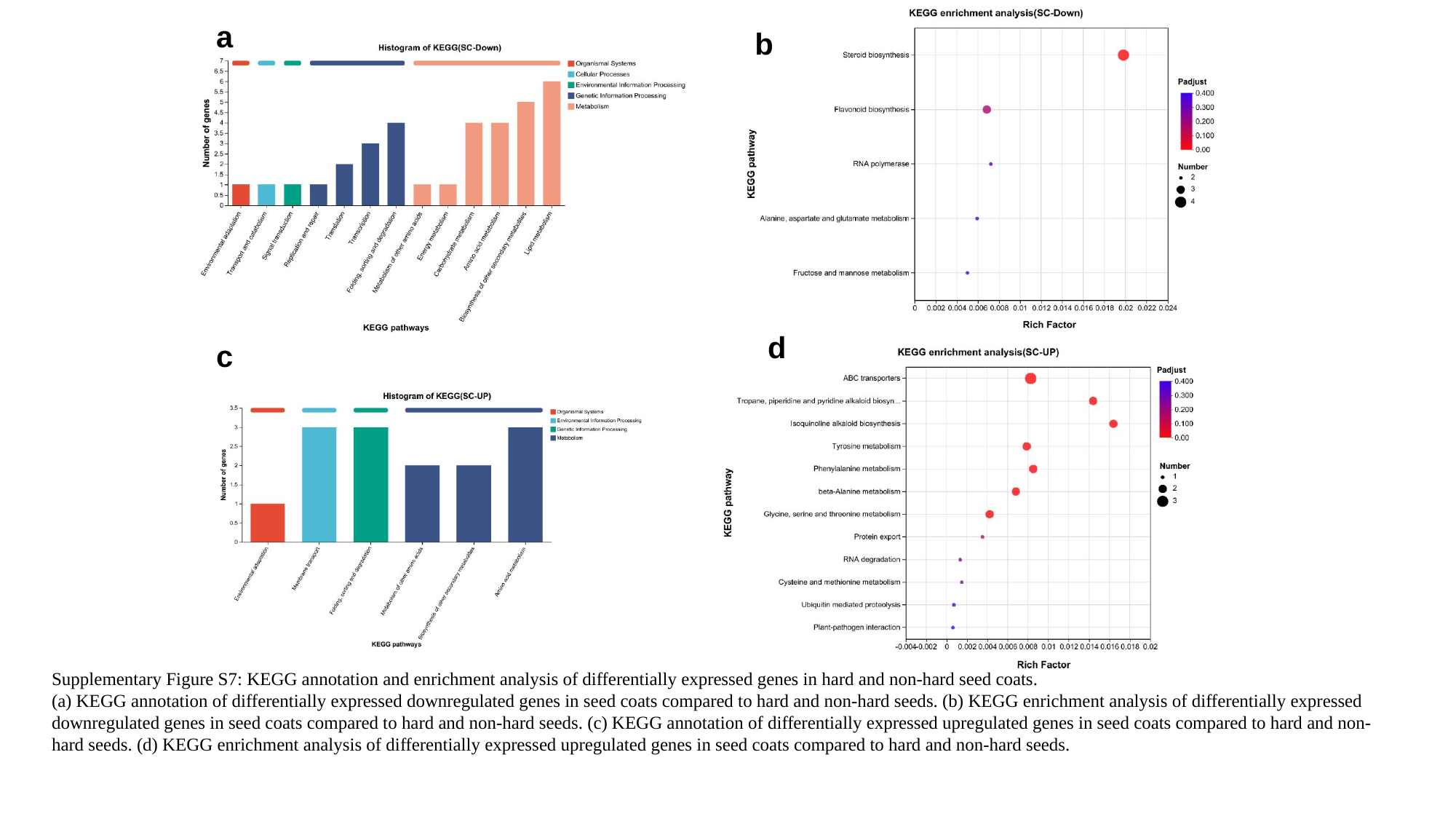

a
b
d
c
Supplementary Figure S7: KEGG annotation and enrichment analysis of differentially expressed genes in hard and non-hard seed coats.
(a) KEGG annotation of differentially expressed downregulated genes in seed coats compared to hard and non-hard seeds. (b) KEGG enrichment analysis of differentially expressed downregulated genes in seed coats compared to hard and non-hard seeds. (c) KEGG annotation of differentially expressed upregulated genes in seed coats compared to hard and non-hard seeds. (d) KEGG enrichment analysis of differentially expressed upregulated genes in seed coats compared to hard and non-hard seeds.

## Slide 8
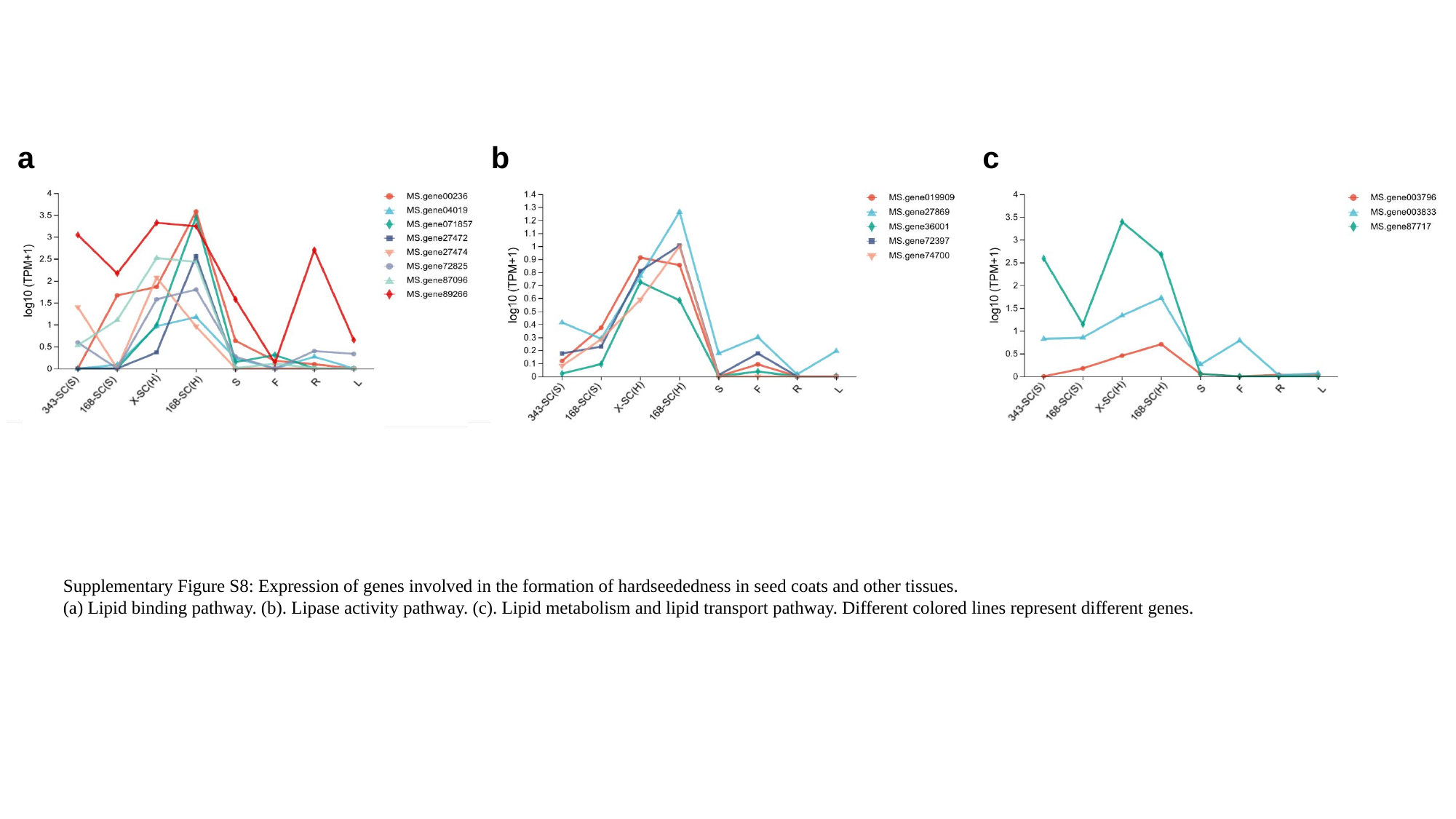

c
b
a
Supplementary Figure S8: Expression of genes involved in the formation of hardseededness in seed coats and other tissues.
(a) Lipid binding pathway. (b). Lipase activity pathway. (c). Lipid metabolism and lipid transport pathway. Different colored lines represent different genes.

## Slide 9
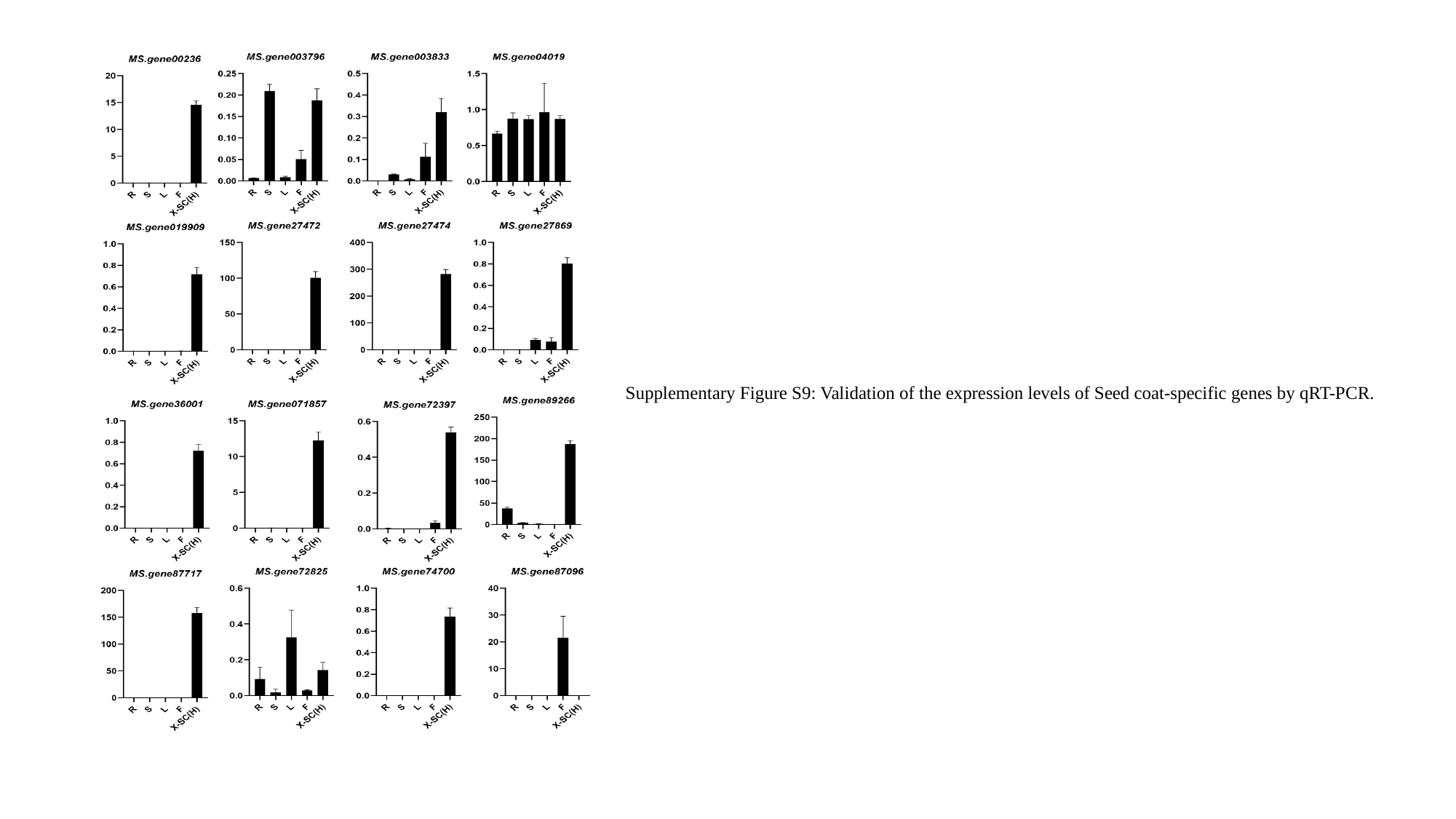

Supplementary Figure S9: Validation of the expression levels of Seed coat-specific genes by qRT-PCR.

## Slide 10
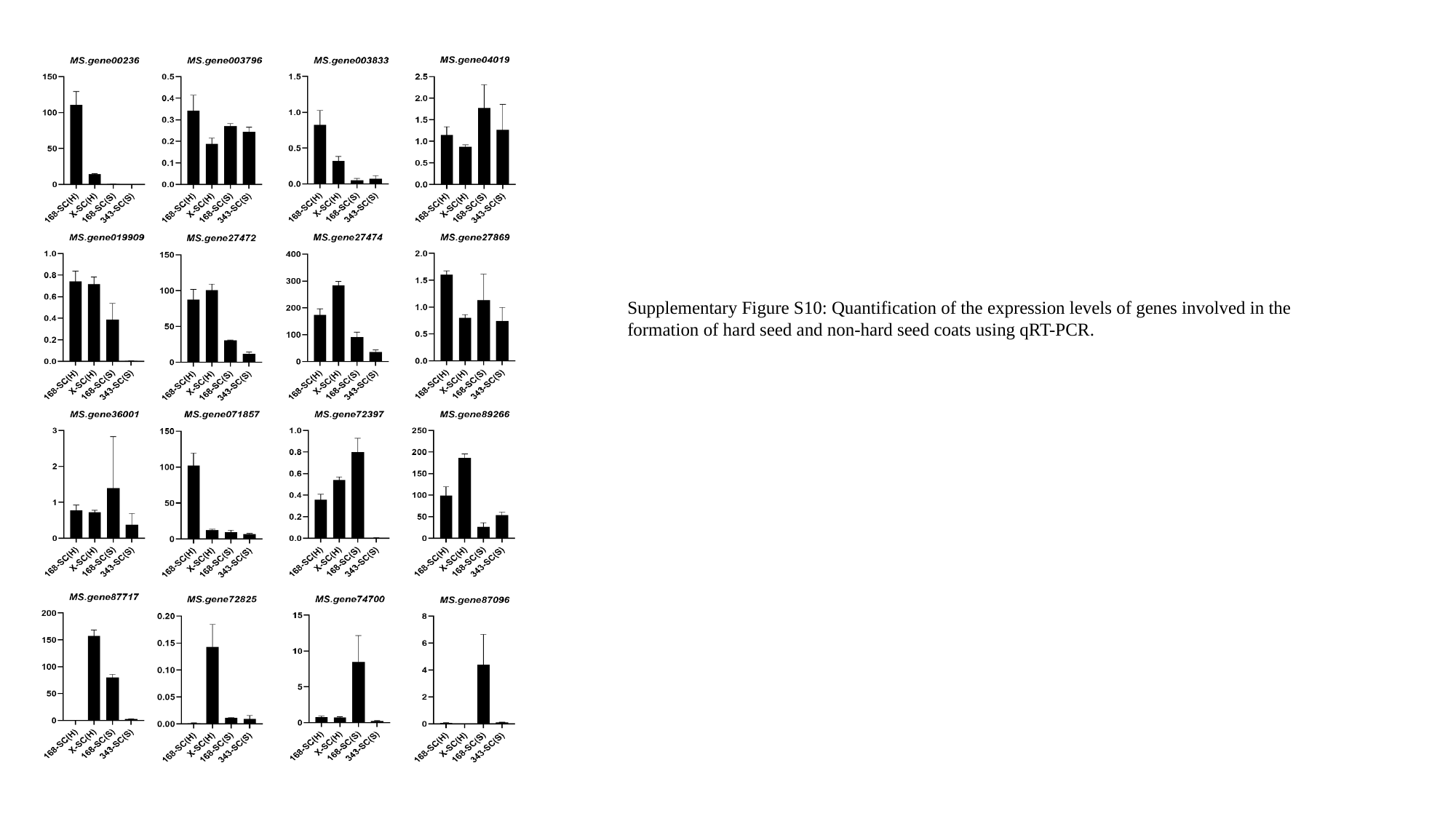

Supplementary Figure S10: Quantification of the expression levels of genes involved in the formation of hard seed and non-hard seed coats using qRT-PCR.
