# Supplementary figures and images for: Global Transcriptome Analysis Reveals the Molecular Mechanism Underlying Seed Physical Dormancy Formation in Medicago sativa
Source: Genes (Basel). 2025 Dec 1;16(12):1438. doi: 10.3390/genes16121438 (PMC12732859; doi:10.3390/genes16121438)

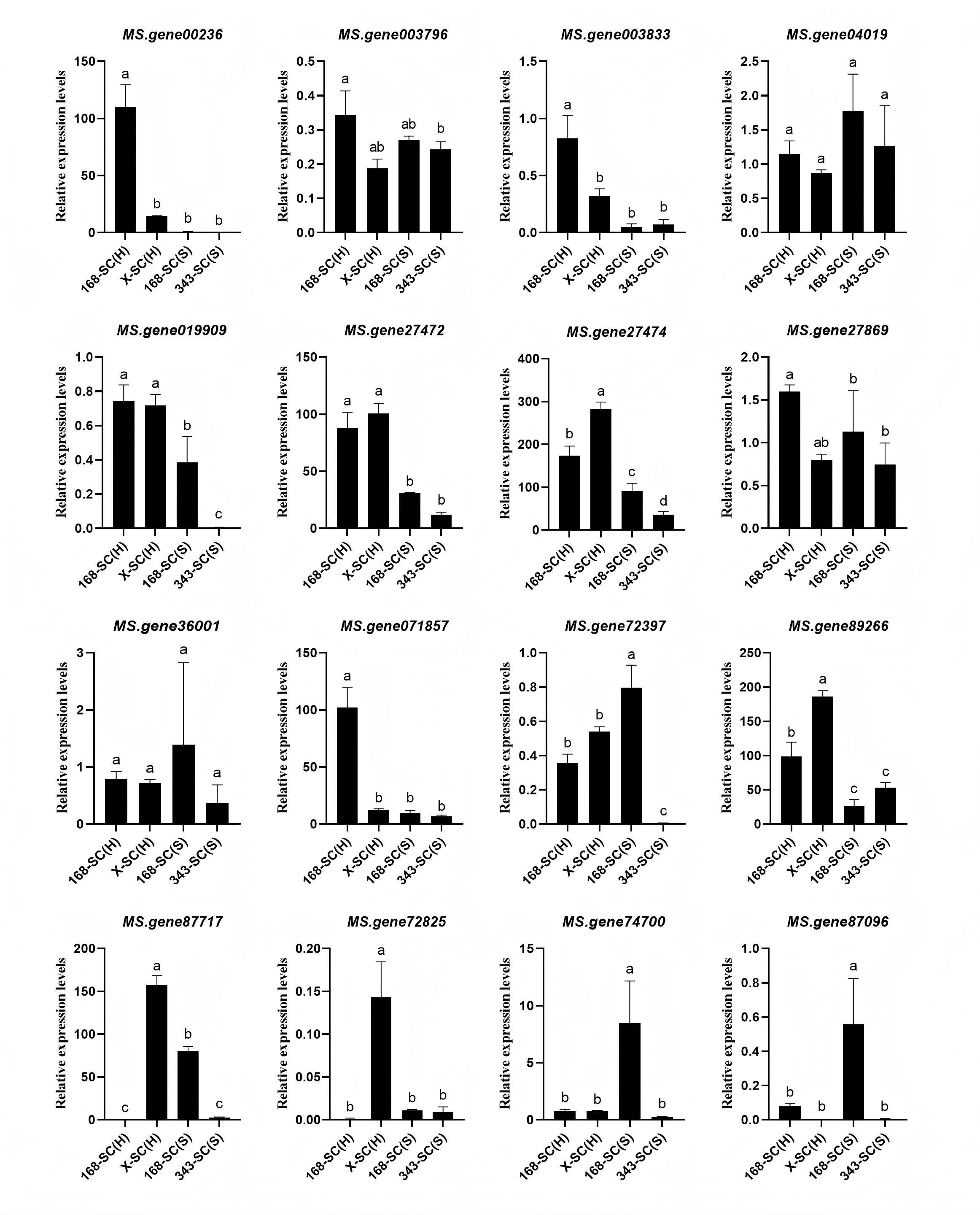

Supplement: Supplementary file 1 [file genes-16-01438-s001.zip › Supplementary Figure S10.jpg]

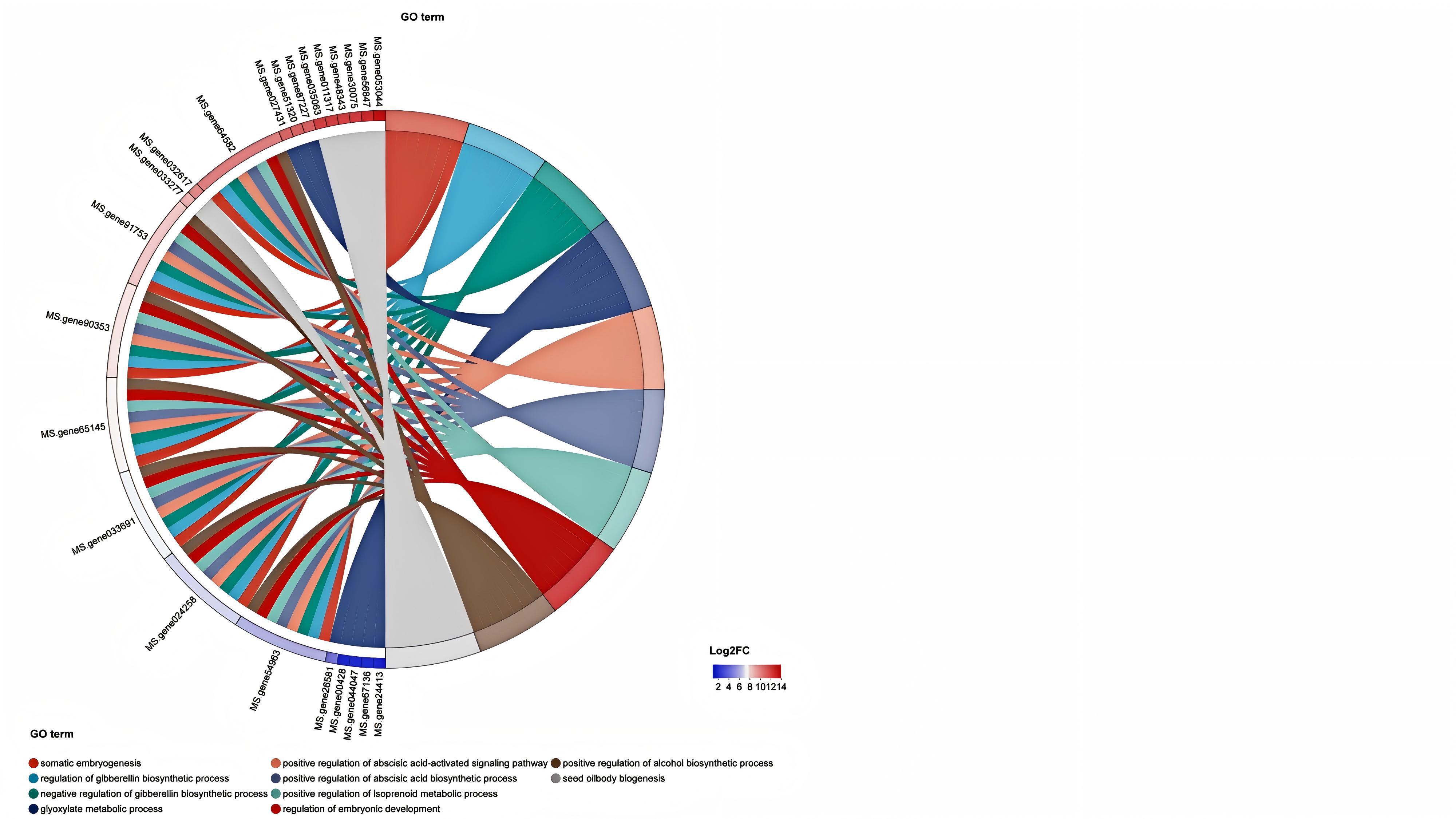

Supplement: Supplementary file 1 [file genes-16-01438-s001.zip › Supplementary Figure S2 .jpg]

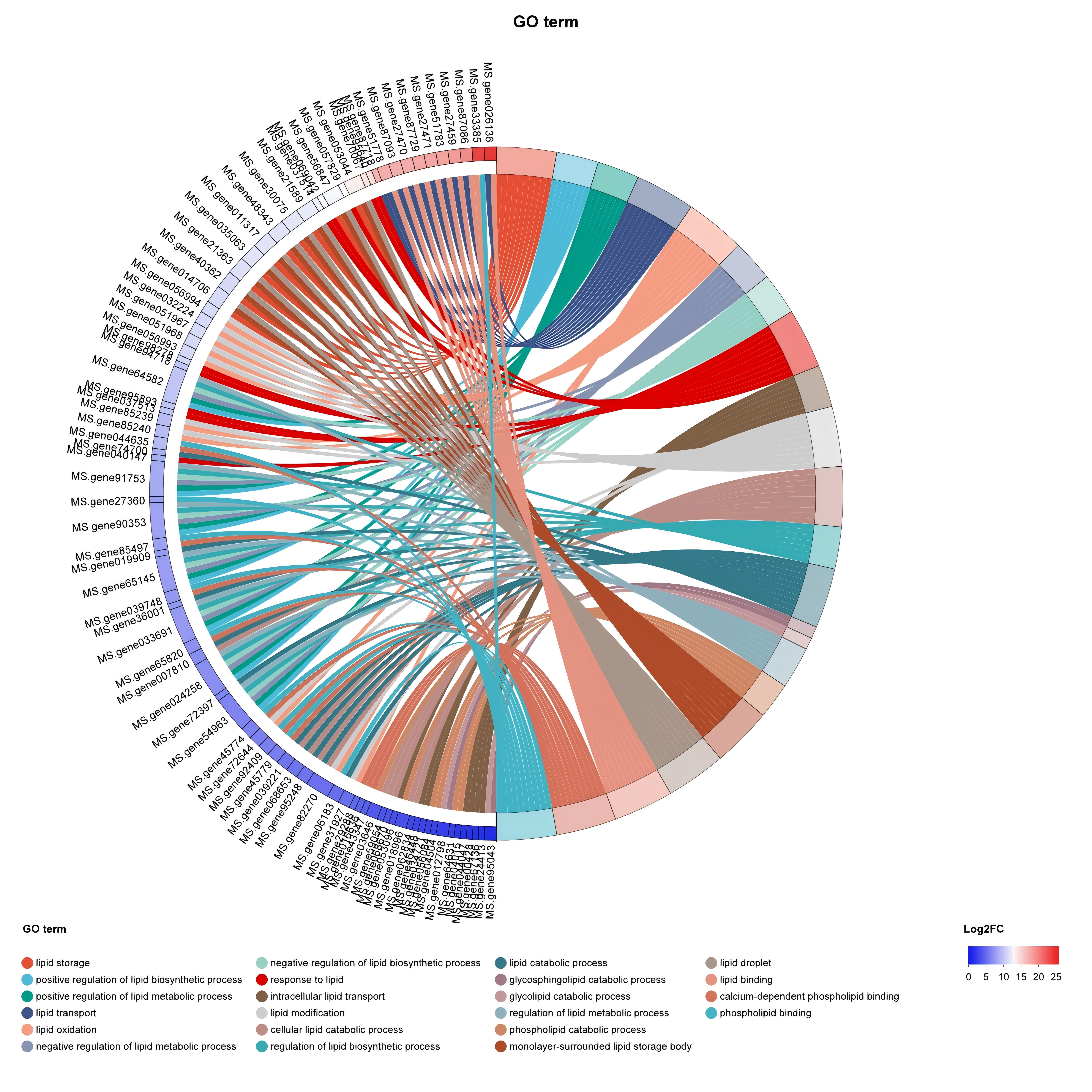

Supplement: Supplementary file 1 [file genes-16-01438-s001.zip › Supplementary Figure S3 .jpg]

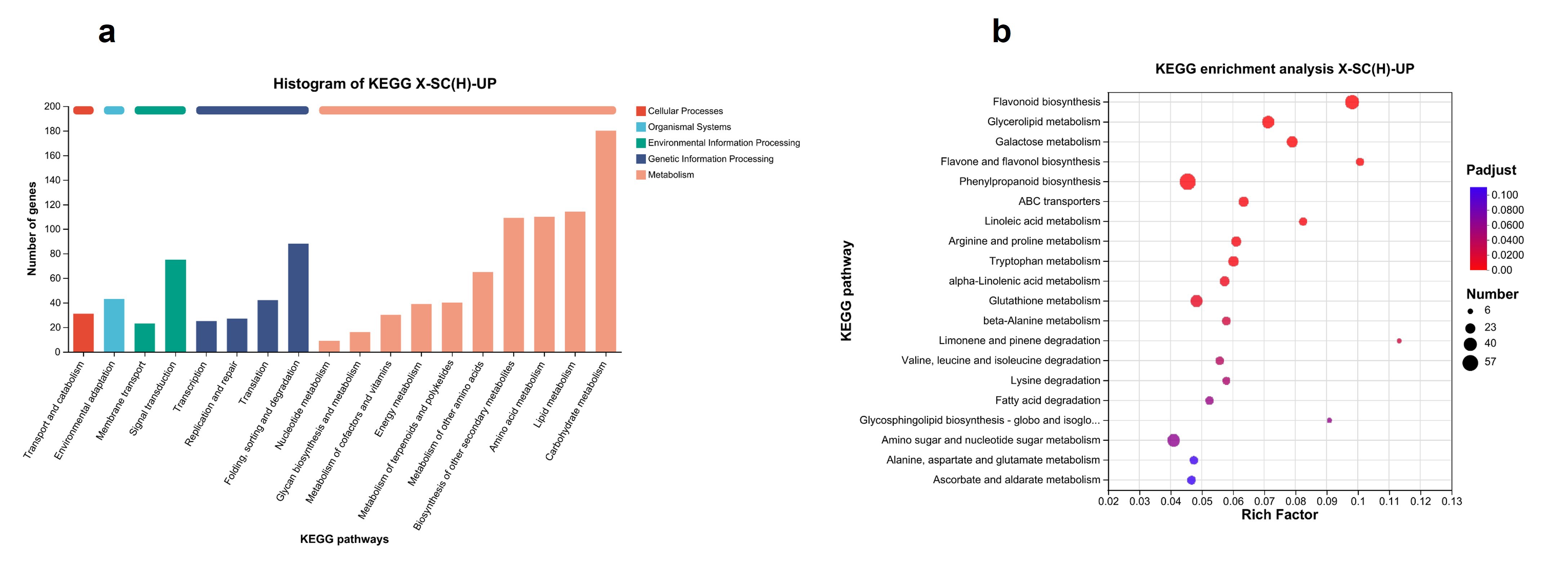

Supplement: Supplementary file 1 [file genes-16-01438-s001.zip › Supplementary Figure S4.jpg]

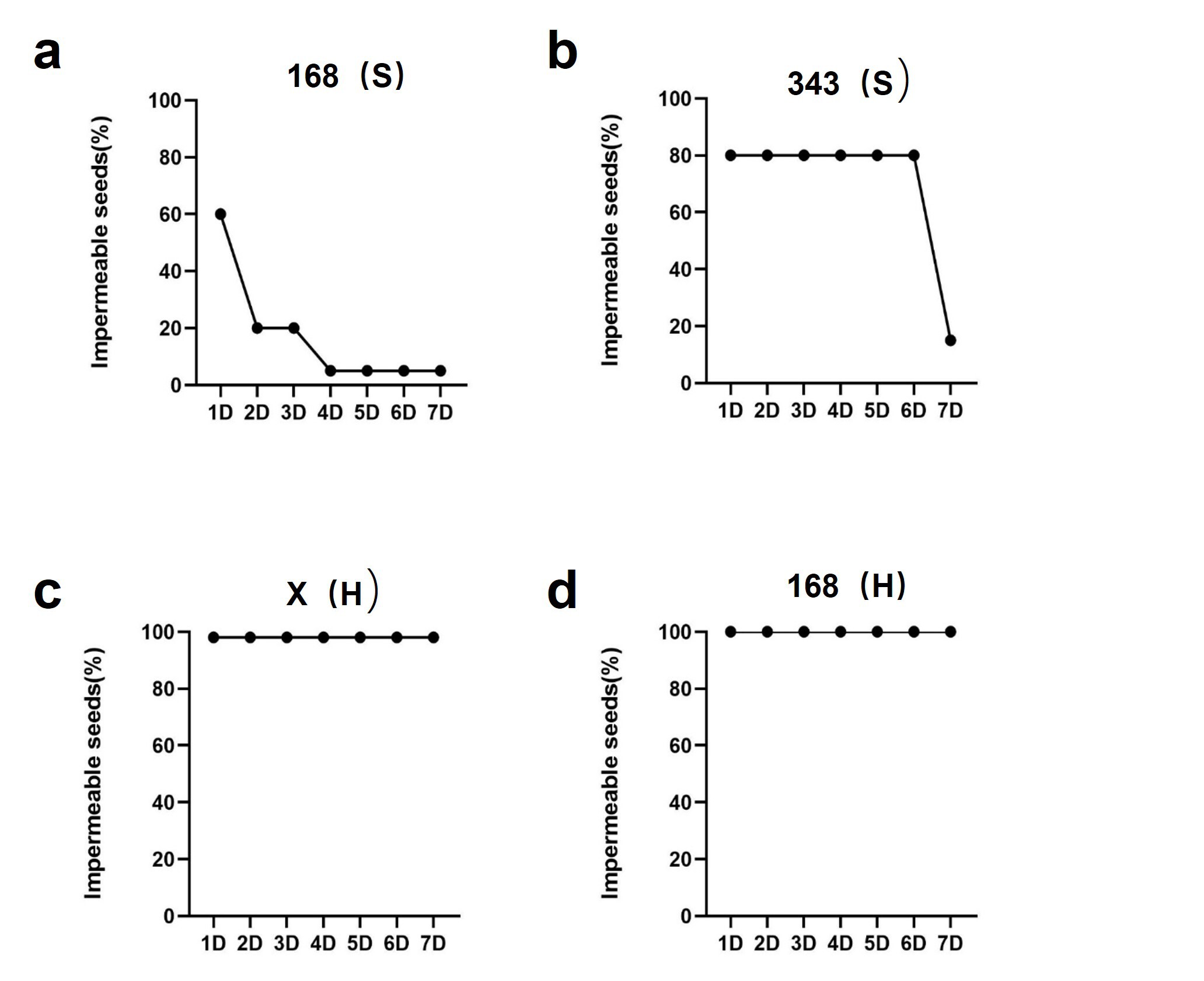

Supplement: Supplementary file 1 [file genes-16-01438-s001.zip › Supplementary Figure S5.jpg]

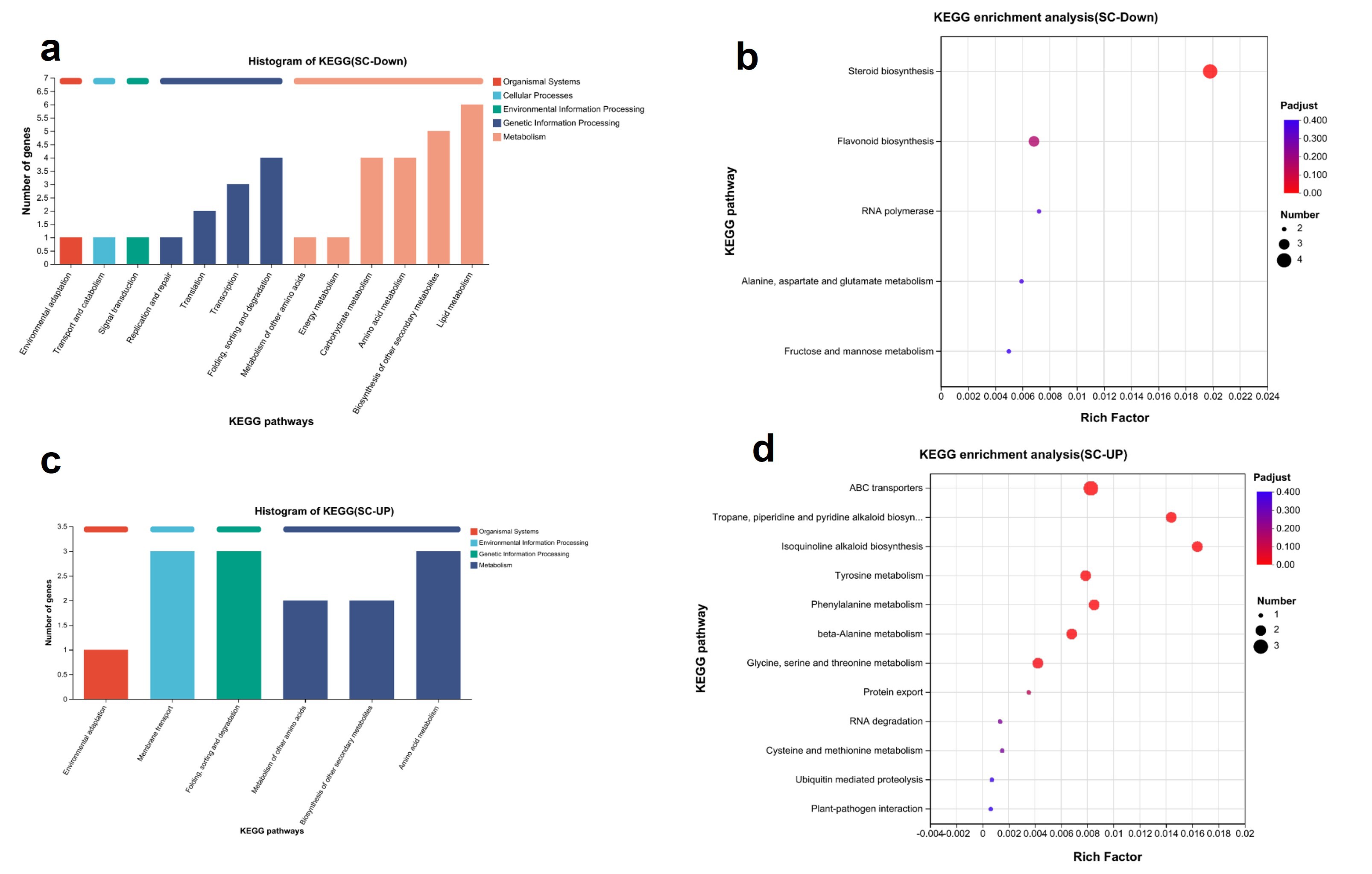

Supplement: Supplementary file 1 [file genes-16-01438-s001.zip › Supplementary Figure S7.jpg]

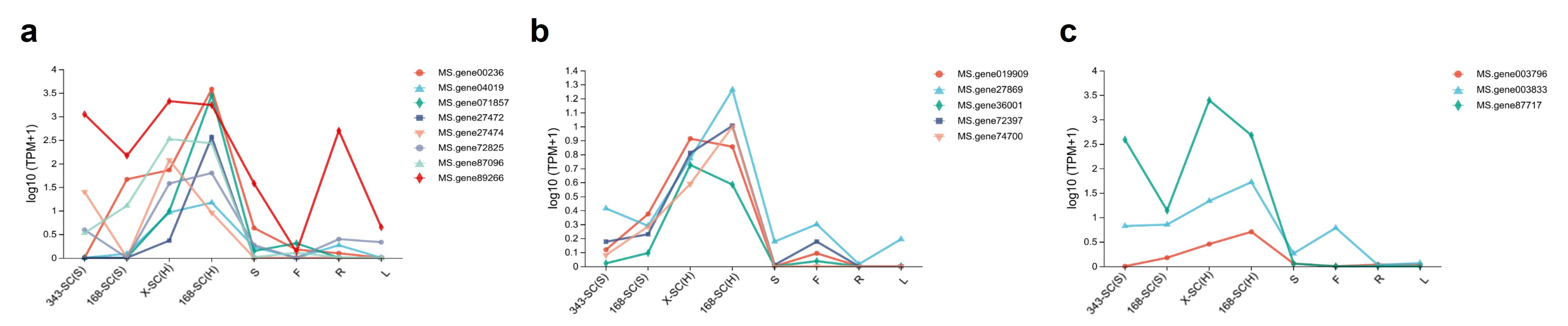

Supplement: Supplementary file 1 [file genes-16-01438-s001.zip › Supplementary Figure S8.jpg]

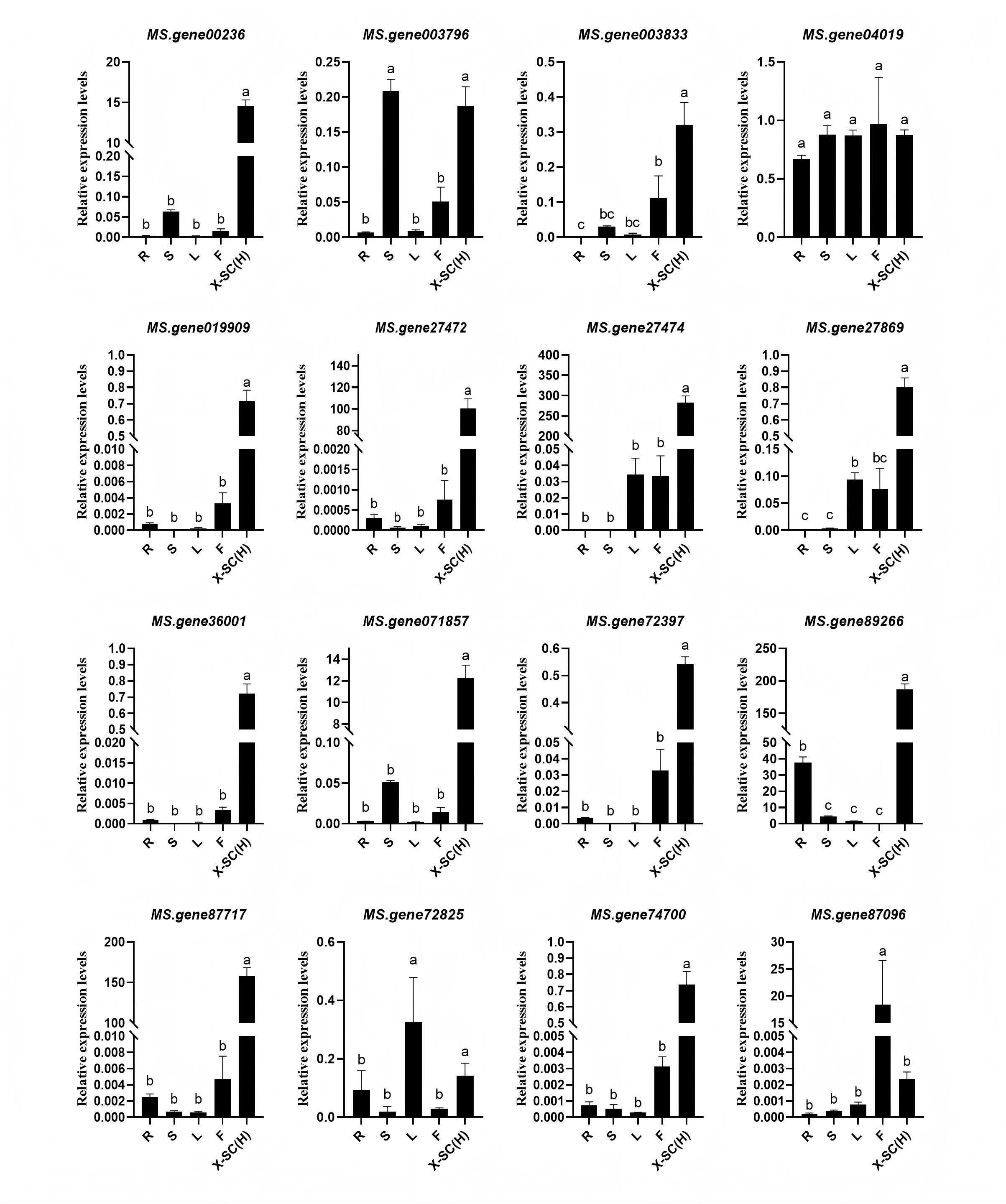

Supplement: Supplementary file 1 [file genes-16-01438-s001.zip › Supplementary Figure S9.jpg]
